# Peripheral Ca_V_2.2 Channels in the Skin Regulate Prolonged Heat Hypersensitivity during Neuroinflammation

**DOI:** 10.1523/ENEURO.0311-24.2024

**Published:** 2024-11-19

**Authors:** Anne-Mary N. Salib, Meredith J. Crane, Amanda M. Jamieson, Diane Lipscombe

**Affiliations:** ^1^Departments of Neuroscience, Brown University, Providence, Rhode Island 02912; ^2^Molecular Microbiology and Immunology, Brown University, Providence, Rhode Island 02912; ^3^Carney Institute for Brain Science, Brown University, Providence, Rhode Island 02912

**Keywords:** chronic pain, hypersensitivity, inflammatory cytokines, neuroinflammation, nociception, peripheral sensitization, voltage-gated calcium channels

## Abstract

Neuroinflammation can lead to chronic maladaptive pain affecting millions of people worldwide. Neurotransmitters, cytokines, and ion channels are implicated in neuroimmune cell signaling, but their roles in specific behavioral responses are not fully elucidated. Voltage-gated Ca_V_2.2 channel activity in skin controls rapid and transient heat hypersensitivity induced by intradermal (i.d.) capsaicin via IL-1ɑ cytokine signaling. Ca_V_2.2 channels are not, however, involved in mechanical hypersensitivity that developed in the i.d. capsaicin animal model. Here, we show that Ca_V_2.2 channels are also critical for heat hypersensitivity induced by i.d. complete Freund adjuvant (CFA). i.d. CFA, a model of chronic neuroinflammation, involves ongoing cytokine signaling for days leading to pronounced edema and hypersensitivity to sensory stimuli. Peripheral Ca_V_2.2 channel activity in the skin was required for the full development and week-long time course of heat hypersensitivity induced by i.d. CFA, but paw edema and mechanical hypersensitivity were independent of Ca_V_2.2 channel activity. CFA induced increases in several cytokines in hindpaw fluid including IL-6 which was also dependent on Ca_V_2.2 channel activity. Using IL-6–specific neutralizing antibodies in vivo, we show that IL-6 contributes to heat hypersensitivity and that neutralizing both IL-1ɑ and IL-6 was even more effective at reducing the magnitude and duration of CFA-induced heat hypersensitivity. Our findings demonstrate a functional link between Ca_V_2.2 channel activity and the release of IL-6 in the skin and show that Ca_V_2.2 channels have a privileged role in the induction and maintenance of heat hypersensitivity during chronic forms of neuroinflammation in the skin.

## Significance Statement

Neuroinflammation can lead to chronic maladaptive pain. Neurotransmitters, ion channels, and cytokines are implicated in neuron-immune signaling, but their importance in mediating specific behavioral responses is not fully elucidated. We show that the activity of peripheral Ca_V_2.2 ion channels in skin plays a unique role in the induction and maintenance of heat hypersensitivity in the complete Freund adjuvant (CFA) model of prolonged neuroinflammation, but not in the development of edema and mechanical hypersensitivity. Blocking peripheral Ca_V_2.2 channel activity reduces local cytokine levels in hindpaws injected with CFA including IL-6, and neutralizing IL-6 reduces CFA-induced heat hypersensitivity. Our studies define key signaling molecules that act locally in the skin to trigger and maintain heat hypersensitivity during chronic neuroinflammation.

## Introduction

Neuroinflammation and peripheral nerve injury can lead to chronic pain, a condition that affects one in five adults in the United States (Kuehn, 2018). Chronic pain is commonly associated with ongoing neuroinflammation, a process that can precede certain neurodegenerative diseases (Amor et al., 2010; W. Zhang et al., 2023). The identity of proteins and signaling molecules that trigger and maintain chronic neuroinflammation may inform the development of therapies with improved specificity and efficacy (McGowan et al., 2009; Hung et al., 2017; Ebersberger, 2018; Sommer et al., 2018).

The responsiveness of nerve endings to sensory stimuli can change rapidly within seconds and serve as a warning of potentially damaging insults (Jain et al., 2020). However, prolonged, chronic neuroinflammation is maladaptive and associated with a range of conditions including painful diabetic neuropathy, arthritis, and neurodegeneration (Amor et al., 2010; Dinarello, 2011; Schaible, 2014; X. X. Fang et al., 2022; Caxaria et al., 2023; W. Zhang et al., 2023). Depending on the stimulus intensity and duration, several long-lasting pathological changes can develop including hypersensitivity to sensory stimuli, edema, and increases in proinflammatory cytokines (Louis et al., 1989; Costigan and Woolf, 2000; S. P. Cook and McCleskey, 2002; Scholz and Woolf, 2002; Costigan et al., 2009). A rise in intracellular calcium in sensory nerve endings and other cells including immune and endothelial is an important trigger of the neuroinflammatory cycle in skin. In chronic forms of neuroinflammation, a positive feedback cycle of cytokine release can last for days to weeks. The targets of cytokines and their receptors are essential for cellular and behavioral changes in sensitivity (Louis et al., 1989; Costigan and Woolf, 2000; S. P. Cook and McCleskey, 2002; Scholz and Woolf, 2002; Costigan et al., 2009), and specific inhibitors of cytokine signaling have been shown to interrupt the inflammatory cycle in skin (Ostrowski et al., 2011; Ebbinghaus et al., 2012; Schaible, 2014; Trier et al., 2019; Cavalli et al., 2021).

Ca_V_2.2 channels in sensory neurons play a major role in regulating neuronal excitability including mediating neurotransmitter release from presynaptic termini in the spinal cord and from nerve endings in the skin (White and Cousins, 1998; Jayamanne et al., 2013; Chai et al., 2017; DuBreuil et al., 2021). The use of Ca_V_2.2^−/−^ KO mouse models and highly specific toxins, including ⍵-CgTx MVIIA used clinically to treat otherwise intractable pain, has established the general importance of Ca_V_2.2 channels in the induction and maintenance of pain (S. S. Bowersox et al., 1996; Y. X. Wang et al., 1998, 2000; Atanassoff et al., 2000; Scott et al., 2002; Altier and Zamponi, 2004; Miljanich, 2004; Staats et al., 2004; Snutch, 2005; Wermeling, 2005; McGivern, 2006; Schroeder et al., 2006; Y. Q. Jiang et al., 2013; Khanna et al., 2019). A single dose of ⍵-CgTx MVIIA, applied intradermally together with capsaicin, specifically prevents the rapid development of heat hypersensitivity without affecting the cross-sensitization of mechanoreceptors in the same animals (DuBreuil et al., 2021). IL-1ɑ, an early released proinflammatory alarmin, couples Ca_V_2.2 channel activity to increased excitability of *Trpv1* nociceptors, and IL-1ɑ regulates capsaicin-induced inflammatory hypersensitivity to both heat and mechanical stimuli (Salib et al., 2024). Here, we test if Ca_V_2.2 channels are necessary for the development of heat and mechanical hypersensitivity associated with intradermal complete Freund adjuvant (i.d. CFA; Kanai et al., 2007; Yu et al., 2008; Fehrenbacher et al., 2012; Moy et al., 2018; S. Zhang et al., 2021). CFA, a heat-killed mycobacterium containing an adjuvant, triggers inflammation through a slow release of antigen at the site of injection (Awate et al., 2013) and is associated with pain that mimics features of inflammatory pain in humans, specifically robust and persistent edema coupled with prolonged hypersensitivity to heat, cold, and mechanical stimuli with a time course lasting >7 d (Larson et al., 1986; Stein et al., 1988; Fehrenbacher et al., 2012).

CFA can stimulate the release of several cytokines in the skin, including IL-1α, IL-1β, IL-4, IL-6, LIF, IL-10, TNF-α, IL-33, IFNγ, CXCL10, CCL2, CCL4, and MDC (J. M. Zhang and An, 2007; Summer et al., 2008; Ellis and Bennett, 2013; Hung et al., 2017; A. D. Cook et al., 2018; Extended Data Table 3-1) which act on cellular targets with some degree of redundancy (J. M. Zhang and An, 2007; Wong et al., 2016). Selective inhibition of cytokine action through the use of neutralizing antibodies (Ebersberger, 2018; Raucci et al., 2019; Zeng et al., 2022) or blocking specific cytokine receptors (Sommer et al., 1999; R. X. Zhang et al., 2008; Summer et al., 2008; Andratsch et al., 2009; Dinarello and van der Meer, 2013; Malsch et al., 2014; Pinho-Ribeiro et al., 2017; Ebersberger, 2018) have revealed functional specificity in neuroinflammatory models of pain. For example, IL-1α-β, IL-6, TNF-α, and CCL2 all promote leukocyte infiltration that further perpetuates cytokine production (Woolf et al., 1997; Cunin et al., 2011; Trier et al., 2019; Jain et al., 2020; Perner et al., 2020; Tamari et al., 2021). Sensory neurons are targets of cytokines, and they express cytokine receptors that can be upregulated in response to injury or insult (X. M. Wang et al., 2009; Hollo et al., 2017; Pinho-Ribeiro et al., 2017; Jain et al., 2020), several of which have been implicated in rapid changes in neuronal excitability including characteristic increased sensitivity to sensory stimuli (Cunha et al., 2005; Kanai et al., 2007; Ebbinghaus et al., 2012; Barabas and Stucky, 2013; Malsch et al., 2014; D. Fang et al., 2015; Pinho-Ribeiro et al., 2017). In cultured sensory neurons, TNF-α and IL-1β have been shown to upregulate TRPV1 expression, and neutralization of TNF-α and IL-1β in vivo reduces thermal hyperalgesia (Schaible, 2014), consistent with their involvement in neuroimmune underlying thermal hyperalgesia (Schaible, 2014; Pinho-Ribeiro et al., 2017).

Here, we report that voltage-gated Ca_V_2.2 channels play a critical role in the development of heat hypersensitivity in CFA-induced neuroinflammation in the skin. We screened 20 cytokines and showed that IL-6 levels depend on Ca_V_2.2 channel activity based on two independent cytokine assay platforms, and we linked its presence directly to CFA-induced heat hypersensitivity.

## Materials and Methods

All mice used were bred at Brown University, and all protocols and procedures were approved by the Brown University Institutional Animal Care and Use Committee (IACUC). All experiments were performed in accordance with approved IACUC protocols. Three- to six-month-old male and female mice were used in all experiments, unless otherwise specified. Experimenters were blind to animal genotype, experimental condition, and solution injected and were only unblinded postanalysis. The Ca_V_2.2^−/−^ global deletion (Ca_V_2.2^−/−^ KO) mouse strain used in these studies (*Cacna1b*^tm5.1DiLi^; MGI) contains a STOP cassette in frame, in Exon 1 of *Cacna1b*, and has been described previously (DuBreuil et al., 2021; Salib et al., 2024). Control mice for comparison to the Ca_V_2.2^−/−^ KO strain were either littermate controls (in all immunophenotyping experiments) or wild-type mice which have been bred in-house in parallel with and from the same genetic background as Ca_V_2.2^−/−^ KO.

### Hindpaw interstitial fluid extraction

Mice were anesthetized using isoflurane (2.0–3.5%) and O_2_ (0.6–0.8 LPM) administered continuously via nosecone for the entire hindpaw fluid extraction process. The plantar surface of the footpad was injected with 20 µl of 100% CFA (Sigma-Aldrich, catalog #F5881) or 20 µl of 100% CFA + 2 µM ⍵-CgTx MVIIA (Alomone Labs, catalog #C-670) on Day 0 using a 30-gauge insulin needle in the center of one hindpaw. Interstitial fluid was extracted daily for 7 d post-injection from anesthetized mice. Saline lavage was performed by injecting 30 µl of saline in the ipsilateral (injected) paw and the contralateral (uninjected) paw to control for repeated daily lavages. Fluid was pooled for eight animals for each experimental condition (ipsilateral lavages pooled, contralateral lavages pooled). Fluid was dispensed into a labeled, prechilled Eppendorf tube on dry ice and stored at −80°C until used for immunoassay analyses.

### Multiplex bead–based immunoassay (LEGENDplex) fluid analyses

The Custom Mouse Inflammation Panel LEGENDplex (BioLegend, LEGENDplex) protocol was followed according to the manufacturer's recommendations. Data were acquired using an Attune NxT Flow Cytometer. We initially ran pilot experiments using hindpaw fluid samples for all experimental conditions to determine sample dilution factors and to screen for the presence of 20 cytokines using capture beads targeting: GM-CSF, IL-1α, IL-1β, IL-4, IL-6, IL-9, IL-10, IL-12p70, IL-17A, IL-22, IL-23, IL-33, TNF-α, IFN-γ, CCL2, CXCL10, CCL4, CCL5, and LIF. Five of 20 cytokines were detected using the LEGENDplex platform in fluid from CFA-injected hindpaws. Following this initial screen, we selected 12 cytokines for further analyses based on our pilot results and published literature on neuroinflammation in the skin (see Extended Data Table 3-1 for references). The BioLegend LEGENDplex Data Analysis Software Suite (Qognit) was used to determine mean fluorescence intensities and to calculate analyte concentrations using concurrently generated standard curves.

### Electrochemiluminescence spot–based immunoassay (MSD) fluid validation

Following LEGENDplex analyses, the remaining samples were assayed on two custom Meso Scale Discovery (MSD) biomarker panels. All samples underwent the same freeze–thaw frequency and duration cycles. Custom MSD multiplex panels were designed using mouse U-PLEX Biomarker Group 1 markers (TNFα, IL-1β, CCL2, CCL4, CXCL-10, IFNγ, IL-33, IL-10, Il-23, and MDC) and was run according to the manufacturer's instructions using a 16-fold dilution. A custom U-PLEX assay (IL-1α and IL-6) was developed and validated using the U-PLEX Development Pack and Multi-Assay SECTOR Plates from MSD. Each capture antibody (R-PLEX anti-mouse IL-1α and U-PLEX anti-mouse IL-6) was incubated with a U-PLEX–coupled Linker for 30 min at room temperature. The plates were read on MSD QuickPlex SQ 120MM Analyzer within 10 min. To test compatibility, eight-point calibration curves were run in the multiplexed assay as well as each validated singleplex assay, and the results were compared. The nonspecific signals on the plate showed no statistically significant difference to the assay background signal indicating that none of the analytes or detection antibodies bind nonspecifically.

Raw data were analyzed using MSD Discovery Workbench analysis software utilizing a four-parameter logistic model (4 PL) with 1/y^2^ weighting.

### Deep punch biopsy for immunophenotyping

In anesthetized mice, we injected the ipsilateral paw with CFA and the contralateral paw was uninjected. Mice were euthanized using an overdose of isoflurane followed by cervical dislocation 1 or 3 d following injection of CFA, and deep punch biopsies of hindpaws were collected from both CFA-injected (ipsilateral) and uninjected (contralateral) paws using 3 mm punch biopsy tools (MedBlade, two punches/paw). Paw punches were placed in Miltenyi Biotec gentleMACS C-tubes containing 5 ml of RPMI-based enzymatic cocktail kept on ice and containing 5% FBS, 50 U DNase I, and 2 mg/ml collagenase IV. Pooled punch biopsies from four mice corresponded to one biological replicate, with 5–7 biological replicates per condition per time point. Pooled samples were weighed to the nearest 0.0001 g prior to digestion and were placed on a prewarmed shaker at 37°C for 1 h at 250 RPM. Single-cell suspensions were obtained using automated dissociation on a gentleMACS dissociator. Following dissociation, 2 mM EDTA was added to neutralize enzymatic activity. Samples were pulsed in a prechilled centrifuge. Tube contents were filtered through a 70 mm filter placed on a 50 ml conical tube, and the gentleMACS C-tube was rinsed with 10 ml filtered 1× PBS + 0.1% BSA. Cells were pelleted at 1,300 RPM for 10 min, and the supernatant was removed. The pelleted cells were washed with 5 ml of 1× PBS + 0.1% BSA and spun down at 1,300 RPM for 5 min, resuspended in 500 ml 1× PBS + 0.1% BSA, and counted prior to staining. Cells were incubated for 10 min on ice with anti-mouse CD16/CD32 antibody (Table 1) diluted in 1× PBS + 0.1% BSA to block Fc receptors (Andersen et al., 2016). Surface antibodies were diluted in 1× PBS + 0.1% BSA, then added to the cells in the presence of anti-CD16/CD32 antibody, and incubated for 20 min on ice in the dark. Following a wash, cells were incubated with fixable viability dye diluted in 1× PBS for 20 min on ice in the dark. Cells were washed and then fixed with 2% PFA for 15 min on ice in the dark. For intracellular staining, cells were permeabilized with 1× Perm/Wash buffer for 30 min on ice. Intracellular antibodies were diluted in 1× Perm/Wash buffer and incubated with the cells for 30 min on ice. Cells were washed and resuspended in 1× PBS for acquisition on an Attune NxT Flow Cytometer (Thermo Fisher Scientific). Analysis was performed using FlowJo Software v10.9.0. Fluorescence minus one controls were used to set analysis gates.

**Table 1.** Antibodies and reagents used for deep punch biopsy immunophenotyping

Target	Fluor	Clone	Lot	Manufacturer	Catalog #	Concentration (final, v/v)
Pan-cytokeratin	Alexa Fluor 488	AE1/AE3	2654135	Thermo Fisher Scientific	53-9003-82	1:200
IL-6	PerCP-eFluor710	MP5-20F3	2609025	Thermo Fisher Scientific	46-7061-82	1:100
F4/80	eFluor660	BM8	2403287	Thermo Fisher Scientific	50-4801-82	1:100
MHC class II (IA/IE)	APC-eFluor780	M5/114.15.2	2626372	Thermo Fisher Scientific	47-5321-82	1:200
Ly6C	V450	AL-21	6175508	BD Biosciences	560594	1:200
Ly6G	BV605	1A8	B360656, B387251	BioLegend	127639	1:200
CD45	BV711	30-F11	B366812	BioLegend	103147	1:200
IL-1α	PE	ALF-161	2326129	Thermo Fisher Scientific	12-7011-81	1:100
CD207	PE-Dazzle 594	4C7	B320091, B394451	BioLegend	144211	1:200
Pro-IL-1β	PE-Cy7	NJTEN3	2396754	Thermo Fisher Scientific	25-7114-82	1:200
Anti-CD16/CD32	Purified	93	101302	BioLegend	101302	1:200
Fixable viability dye	eFluor506	n/a	2290921	Thermo Fisher Scientific	65-0866-14	1:1,000
Super Bright staining buffer	n/a	n/a	2633469	Thermo Fisher Scientific	SB-4401-42	1:200
Staining buffer	1× PBS + 0.1% BSA (made in-house)					
2% PFA	4% PFA (made in-house) diluted in 1× PBS					
BD Perm/Wash buffer 10× solution	diluted to 1× in dH20	n/a	2028447	BD Biosciences	51-2091KZ	

A list of cell surface and intracellular cytokine antibodies used for flow cytometry analysis of cells isolated from deep hindpaw punch biopsies (Fig. 2 and Extended Data Fig. 2-1).

### Behavioral assessments

The experimenter was blind to genotype and experimental conditions for all behavioral experiments. Hindpaw withdrawal responses to radiant heat were elicited from mouse hindpaws using the Hargreaves method (Plantar Analgesia Meter, IITC Life Science). Mice were placed in plexiglass boxes on an elevated glass plate and allowed to habituate for 30 min prior to testing. A radiant heat source was positioned beneath the mice and aimed using low-intensity visible light to the plantar surface of the hindpaw. For all trials, laser settings were as follows: idle intensity at 5% and active intensity at 50% of maximum. The cutoff time was 30 s. Trials began once the high-intensity light source was activated and ended once the mouse withdrew, shook, and/or licked their hindpaws following stimulation. Immediately upon meeting the response criteria, the high-intensity light source was turned off. The response latency was measured to the nearest 0.01 s for each trial using the built-in timer corresponding to the duration of the high-intensity beam. Three trials were conducted on each hindpaw for each mouse, and trials were counterbalanced, with alternating measurements from the ipsilateral and contralateral paws with at least 1 min rest between trials (Hargreaves et al., 1988). An average of three trials were used for the analysis. *N* values reported are the number of mice. After baseline measures, mice were anesthetized with isoflurane during all intradermal injections. Daily behavior was assessed at the same time of day, and uninjected paws were also assessed daily as a control for exposure to anesthetics and repeated measures.

Mechanical paw withdrawal responses were elicited by an automated von Frey plantar aesthesiometer (catalog #37550, Ugo Basile). Mice were placed in an elevated plexiglass box with a wire mesh bottom and were allowed to habituate for 30 min prior to testing. The plantar surface of hindpaws was assessed using a steady ramp of force ranging from 0 to 8 g for up to 90 s. The trial is automatically terminated when the filament buckles or the paw is withdrawn, and force and reaction time are captured. Three trials were conducted on each hindpaw for each mouse, and trials were counterbalanced with alternating measurements from the ipsilateral and contralateral paw with at least 1 min rest between trials. An average of three trials were used for the analysis. *N* values reported are the number of mice. After baseline measures, mice were anesthetized with isoflurane during all intradermal injections.

### Data quantification and statistical analysis

Statistical analyses were performed Prism (Version 10; GraphPad). All data are presented as the mean ± SE. Post hoc corrections for multiple comparisons were performed when applicable and as indicated in the Results section.

### Data availability statement

The Ca_V_2.2^−/−^ KO mouse strain (Cacna1b^tm5.1DiLi^) is described in the MGI database and is available by request from the lab.

## Results

We used the i.d. CFA model of neuroinflammation in mouse hindpaws to establish if there is a direct link between Ca_V_2.2 channel activity and the release of specific cytokines involved in neuroimmune signaling and behavioral changes in sensitivity to heat and mechanical stimuli. We monitored the development and maintenance of well-described features of CFA-induced neuroinflammation in the skin by measuring increased sensitivity to heat and mechanical stimulation, increased paw edema, increased immune cell infiltration, and increased levels of cytokines localized to the injected paw. To assess the role of peripheral Ca_V_2.2 channels in neuroinflammation in the skin, we measured responses to i.d. CFA in hindpaws and compared WT mice, global Ca_V_2.2^−/−^ knock-out mice (Ca_V_2.2^−/−^ KO), and WT mice coinjected with the highly specific Ca_V_2.2 blocker ⍵-CgTx MVIIA.

### Ca_V_2.2 channel activity is necessary for prolonged CFA-induced heat hypersensitivity

**Figure 1.**
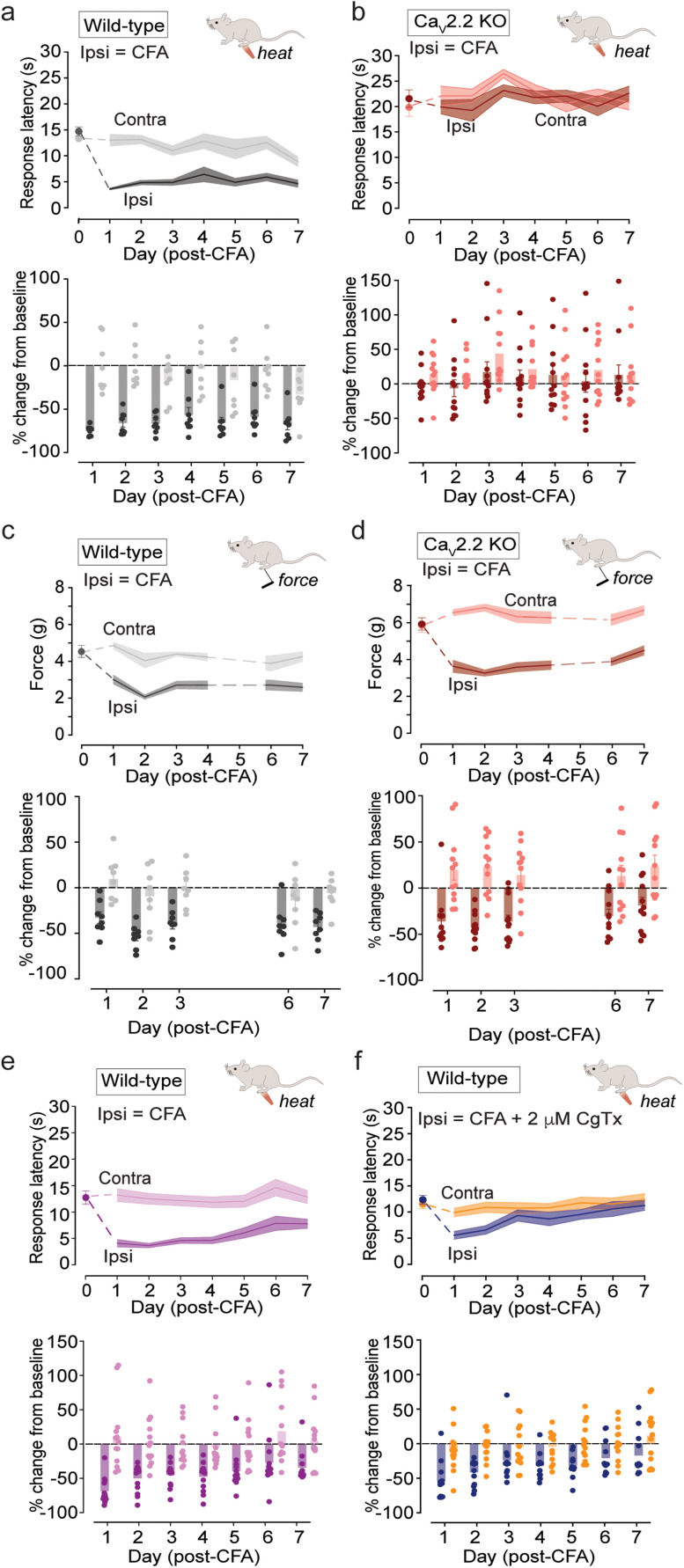
Ca_V_2.2 channel activity is necessary for the development of heat, but not mechanical hypersensitivity triggered by i.d. CFA. Daily response latencies to radiant heat (***a***, ***b***, ***e***, ***f***) and withdrawal thresholds to mechanical stimuli (***c***, ***d***) for contralateral (uninjected) and ipsilateral (CFA injected) hindpaws before (Day 0) and for 7 d post i.d. 20 μl CFA are shown as mean (solid line; top) ± SE (shaded area; top) and as individual measurements shown as percentage change from baseline (solid circles; bottom) and mean ± SE (shaded bars; bottom). Data are shown for two experimental conditions: WT (***a***, ***c***: gray, *n* = 8) compared with Ca_V_2.2^−/−^ KO (***b***, ***d***: red, *n* = 12) mice and WT CFA (***e***: magenta, *n* = 13) compared with WT CFA coinjected with 2 μM ⍵-CgTx MVIIA [***f***: blue (ipsi), orange (contra), *n* = 13]. ***a***, ***b***, Statistical comparison of % change in withdrawal response latencies to radiant heat for CFA by two-way ANOVA with Tukey HSD correction for multiple comparisons and repeated measures: *p*(WT ipsi | WT contra) = 0.0001; *p*(WT ipsi | KO ipsi) = 0.0232; *p*(KO ipsi | KO contra) = 0.8796. Significance for time | genotype interaction = *p* < 0.0001. ***c***, ***d***, Same mice used in ***a*** and ***b***. Statistical comparison of % change in withdrawal thresholds to mechanical stimuli from baseline following CFA by ANOVA with Tukey HSD correction for multiple comparisons and repeated measures: *p*(WT ipsi | WT contra) =0.0073; *p*(WT ipsi | KO ipsi) = <0.0001; *p*(KO ipsi | KO contra) = <0.0001. Significance for time | genotype interaction *p* = 0.0008. The baseline sensitivities between WT and Ca_V_2.2^−/−^ KO mice to heat and mechanical stimulation are consistently different. For heat, ipsi on Day 0: WT versus KO, *p* value = 0.0084; analysis of variance using a repeated measures two-way ANOVA followed by post hoc tests with Tukey multiple-comparisons correction. For mechanical, ipsi on Day 0: WT versus KO, *p* value = 0.0477; two-way ANOVA with Tukey HSD correction for multiple comparisons. ***e***, ***f***, Statistical comparisons of withdrawal latencies to radiant heat following CFA by ANOVA with Tukey HSD correction for multiple comparisons and repeated measures: time | injection CFA: *p* = 0.0100; time | injection CFA + CgTx MVIIA: *p* = 0.2948. Day 3: *p*(WT CFA ipsi | contra) *p* = 0.0002, mean = −42.9%; *p*(WT CFA + CgTx MVIIA ipsi | contra) *p* = 0.116, mean = −19.9%.

Baseline behavioral responses to heat and mechanical stimuli applied to hindpaws were measured on Day 0 prior to i.d. CFA and then daily for 1 week (Fig. 1). WT mice exhibited characteristic increases in sensitivity to heat (Fig. 1*a*) and mechanical (Fig. 1*c*) stimuli within 1 d of i.d. CFA, as compared with contralateral paw responses. We observed shorter response latencies to radiant heat and lower response thresholds required to elicit paw withdrawal to mechanical stimuli following i.d. CFA, which persisted for the 7 d period that we monitored behavior.

**Figure 2.**
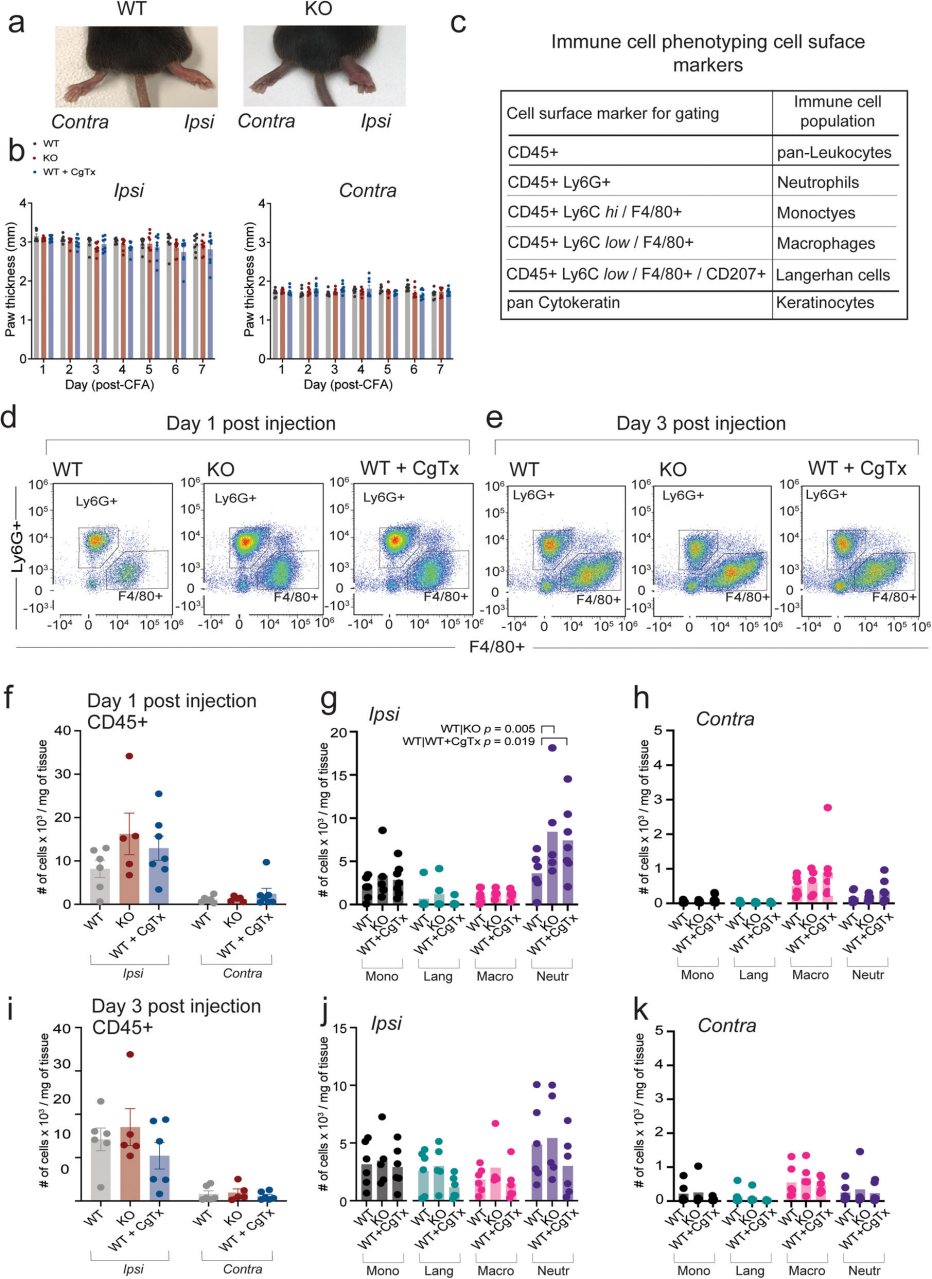
Edema and leukocyte infiltration following intradermal (i.d.) CFA is independent of Ca_V_2.2 channel activity. ***a***, Example images of WT and Ca_V_2.2^−/−^ KO mice paws 1 d after CFA injection. ***b***, Paw thickness measured daily using digital calipers. Serial edema measurements of contralateral (control) and ipsilateral (CFA injected) hindpaws of mice measured daily for 1 week following 20 μl i.d. injection of CFA in wild type (WT, gray), CFA in Ca_V_2.2^−/−^ (KO, red), and CFA in WT coinjected with 2 μM ⍵-CgTx MVIIA (WT + CgTx, blue). *N* = 8 mice for each condition. Average values of 1.74 ± 0.02 mm for contralateral paws and 2.95 ± 0.03 mm ipsilateral paws. There were no statistical differences in paw thickness of CFA injected paws among the three conditions analysis of variance using two-way ANOVA with for (WT/KO/CgTx CFA ipsi paw thickness) | time interaction *p* = 0.6633. ***c***, Immunophenotyping of leukocytes recruited to the hindpaw following CFA injection using a multicolor flow cytometry panel comprising cell surface markers used to identify leukocyte populations (Table 1 and Materials and Methods). ***d–k***, Two deep punch biopsies were collected from each hindpaw, from each animal, 1 and 3 d post-CFA; samples were pooled (4 pooled ipsilateral paws, 4 contralateral paws per biological replicate; 5–7 biological replicates per condition). Cell counts were normalized to the starting weight of pooled predigested tissue. ***d***, ***e***, Representative flow cytometry plots showing increased neutrophils (Ly6G+) 1 d after CFA injection in the absence of Ca_V_2.2 channel activity when compared with WT controls. Representative flow cytometry plots showing no difference in neutrophils (Ly6G+) 3 d after CFA injection across all three experimental conditions. ***f***, ***i***, CD45+ leukocyte infiltration is increased in ipsilateral (CFA injected) paw tissue on days 1 and 3 compared with uninjected contralateral paw tissue in wild type (WT), Ca_V_2.2^−/−^ (KO), and WT coinjected with 2 μM ⍵-CgTx MVIIA (WT + CgTx). Analysis of variance using a repeated measures two-way ANOVA, followed by post-hoc tests with Tukey multiple-comparisons correction. Day 1: *p*(WT ipsi | WT contra CD45+) = 0.0414; *p*(KO ipsi | KO contra CD45+) = 0.0002; *p*(WT CFA + CgTx MVIIA ipsi | contra CD45+) = 0.0017. Day 3: *p*(WT ipsi | WT contra CD45+) = 0.0006; *p*(KO ipsi | KO contra CD45+) = 0.0002; *p*(WT CFA + CgTx MVIIA ipsi | contra CD45+) = 0.0079. Ca_V_2.2 channel activity does not impact leukocyte infiltration into hindpaw tissue following i.d. CFA on Day 1 or Day 3; levels of leukocytes in ipsilateral paw tissue are not different when compared across conditions. Day 1: *p*(WT ipsi | KO ipsi CD45+) = 0.0665; *p*(KO ipsi | WT CFA + CgTx MVIIA ipsi CD45+) = 0.5953; *p*(WT ipsi | WT CFA + CgTx MVIIA ipsi CD45+) = 0.3014. Day 3: *p*(WT ipsi | KO ipsi CD45+) = 0.6876; *p*(KO ipsi | WT CFA + CgTx MVIIA ipsi CD45+) = 0.1458; *p*(WT ipsi | WT CFA + CgTx MVIIA ipsi CD45+) = 0.4829. ***g***, ***h***, Mean cell counts for each cell population 1 d after CFA injection; 1 d following CFA injection Ca_V_2.2 KO and mice coinjected with CgTx MVIIA recruit more neutrophils to the injury site when compared with WT mice injected with CFA alone. Mean neutrophil count normalized to starting weight of the punch: Day 1 WT = 3,620 ± 529 neutrophils/mg of starting tissue; KO = 8,424 ± 1,365 neutrophils/mg of starting tissue; WT + CgTx MVIIA = 7,414 ± 670 neutrophils/mg of starting tissue; analysis of variance using two-way ANOVA with Tukey HSD correction for multiple comparisons; *p*(WT CFA ipsi | KO CFA ipsi) = 0.00054; *p*(WT CFA ipsi | WT CFA + CgTx ipsi) = 0.0191; *p*(KO CFA ipsi | WT CFA + CgTx ipsi) = 0.7612. ***j***, ***k***, Mean cell count for each cell population 3 d after CFA injection; no significant difference across cell populations, and specifically neutrophils analysis of variance using two-way ANOVA with Tukey HSD correction for multiple comparisons on Day 3 *p*(WT CFA ipsi | KO CFA ipsi) = 0.9080; *p*(WT CFA ipsi | WT CFA + CgTx ipsi) = 0.3256; *p*(KO CFA ipsi | WT CFA + CgTx ipsi) = 0.1803. See Extended Data Table 2-1 for all statistical analyses of cell types in CFA-injected ipsilateral paw.

10.1523/ENEURO.0311-24.2024.f1-1 Figure 1-1 Validation of CFA-induced heat hypersensitivity inhibited by pharmacological block of peripheral Ca_V_2.2 channels in an inbred independent mouse strain. 16–20-week-old C57BL/6J mice were ordered from Jackson Laboratories (Strain # 0000664) and were acclimated at least one week prior to behavioral testing. Under blinded conditions, mice were injected with CFA alone (n = 13), or CFA + 2 μM ⍵-CgTx MVIIA (n = 13) as described previously (see in methods and figure 2. Solid line represents the mean response latency, the shaded area represents standard error. Download Figure 1-1, TIF file.

To establish the contribution of Ca_V_2.2 channels to these behavioral responses, we assessed the effects of i.d. CFA in a Ca_V_2.2 knock-out strain (Ca_V_2.2^−/−^ KO; DuBreuil et al., 2021; Salib et al., 2024) and WT mice injected with i.d. ⍵-CgTx MVIIA (Fig. 1). Baseline paw withdrawal response times in Ca_V_2.2^−/−^ KO mice were longer (radiant heat) and occurred at higher thresholds (mechanical) compared with WT (Fig. 1*b,d*. For heat, Day 0: WT vs KO, *p* value = 0.0084, two-way ANOVA with Tukey HSD correction for multiple comparisons. For mechanical, Day 0: WT vs KO, *p* value = 0.0477, two-way ANOVA with Tukey HSD correction for multiple comparisons). The reduced baseline sensitivity to sensory stimuli in Ca_V_2.2^−/−^ KO mice is expected because synaptic transmission is reduced in Ca_V_2.2 channel-lacking presynaptic nerve endings in the spinal cord (DuBreuil et al., 2021). To normalize this difference in baseline responses, we also show changes in sensitivity to heat and mechanical stimulation as a percentage change from baseline. Within 1 d following i.d. CFA, ipsilateral paws of Ca_V_2.2^−/−^ KO mice developed edema (Fig. 2*a,b*) and increased sensitivity to mechanical stimulation based on percentage change from baseline, which were not distinguishable from WT (Fig. 1*c,d*). By comparison, the sensitivity of Ca_V_2.2^−/−^ KO mice to radiant heat did not change following CFA during the 7 d assessment period (*p* = 0.8796; Fig. 1*b*, analysis of variance using a repeated measures two-way ANOVA, followed by post-hoc tests with Tukey multiple-comparisons correction). These data, combined with previous studies using the capsaicin model of rapidly developing and transient heat hypersensitivity (DuBreuil et al., 2021; Salib et al., 2024), show that Ca_V_2.2 channels have a privileged and specific role in neuroimmune signaling that triggers long-lasting heat hypersensitivity in the skin. In contrast, CFA-induced mechanical hypersensitivity and paw edema developed and were maintained by signaling pathways that were independent of Ca_V_2.2 channel activity.

10.1523/ENEURO.0311-24.2024.f2-1 Figure 2-1 Gating strategy for hind paw deep punch biopsy immunophenotyping flow cytometry analyses (shown in Fig. 2) Analyses were performed using FlowJo Software v10.9.0. Housekeeping exclusion of cell doublets, dead cells, debris, and red blood cells. Cells were assigned an identity based on expression of specific cell surface markers using antibodies with distinct fluorophores. The cell surface marker CD45 was used to identify pan-leukocytes, that were further characterized into individual cell populations based on the expression of additional cell surface markers: Neutrophils express Ly6G, Langerhans cells express F4/80 and CD207, Monocytes express F4/80 and high expression of Ly6C, and Macrophages express F4/80 and low expression of Ly6C. For each leukocyte population, we analyzed intracellular cytokine levels of IL-6, IL-1β, and IL-1α using intracellular cytokine antibodies (See Table 1 and Methods). Download Figure 2-1, TIF file.

10.1523/ENEURO.0311-24.2024.t2-1 Table 2-1 Statistical analysis of variance using 2-way ANOVA with Tukey HSD correction for multiple comparisons across conditions, cell types, and days, with adjusted p-values. Download Table 2-1, DOC file.

### Inhibiting peripheral Ca_V_2.2 channels reduces the amplitude and duration of CFA-induced heat hypersensitivity

To assess the importance of Ca_V_2.2 channels in skin, distinct from their role in supporting synaptic transmission at sensory presynaptic sites in the dorsal horn of the spinal cord, we used the highly specific Ca_V_2.2 channel blocker ⍵-CgTx MVIIA (S. Bowersox et al., 1997; Miljanich, 2004; de Souza et al., 2013; Jayamanne et al., 2013) co-injected together with CFA into hindpaws of WT mice (Fig. 1*f*). Baseline paw withdrawal responses to heat were not different between ipsilateral and contralateral paws [*p*(ipsi | contra) = 0.5220; analysis of variance using a repeated measures two-way ANOVA, followed by post hoc tests with Tukey multiple-comparisons correction; Fig. 1*e,f*]. This confirms that i.d. ⍵-CgTx MVIIA does not reach the spinal cord and does not interfere with the transmission of signals from the periphery to central sites that mediate paw withdrawal (DuBreuil et al., 2021). Intradermal ⍵-CgTx MVIIA did, however, reduce both the magnitude and duration of heat hypersensitivity induced by i.d. CFA (Fig. 1*f*). By Day 3, ⍵-CgTx MVIIA reduced the effects of i.d. CFA significantly compared with control responses [Day 3: WT CFA + CgTx MVIIA (ipsi | contra) *p* = 0.116; Fig. 1*f*. Day 3: WT CFA (ipsi | contra) *p* = 0.0002; Fig. 1*e*. Analysis of variance using a repeated measures two-way ANOVA, followed by post hoc tests with Tukey multiple-comparisons correction]. We also validated the inhibitory effect of ⍵-CgTx MVIIA on CFA-induced heat hypersensitivity in the hindpaws of WT mice using an independent cohort of inbred C57BL/6 mice (Jackson Laboratory #000664; Extended Data Fig. 1-1). These data, combined with previous studies using the i.d. capsaicin model of fast, transient heat hypersensitivity (DuBreuil et al., 2021; Salib et al., 2024) show that peripheral Ca_V_2.2 channels contribute selectively to the development of heat hypersensitivity in the skin during short and prolonged forms of neuroinflammation. Local inhibition of Ca_V_2.2 channels using a single i.d. injection of CgTx MVIIA, coincident with CFA, was sufficient to reduce the magnitude of the early phase and significantly shorten the time course of heat hypersensitivity in this model of neuroinflammation. In contrast, CFA-induced mechanical hypersensitivity and paw edema developed independent of Ca_V_2.2 channel activity.

### CD45+ leukocyte infiltration is independent of Ca_V_2.2 channel activity, but neutrophil density is transiently higher when Ca_V_2.2 channel activity is absent or reduced

We assessed the immune cell composition of hindpaw edema in postmortem deep punch biopsies of CFA-injected ipsilateral and contralateral hindpaws on Days 1 and 3 post i.d. CFA (Fig. 2*c–k*). We analyzed pooled samples from four mice (5–7 biological replicates per condition, 20–28 mice per day per condition) and normalized cell counts to the starting weight of pooled tissue (Fig. 2*f–k*; see Extended Data Fig. 2-1 and Materials and Methods for flow cytometry gating strategy). Deep punch biopsies of hindpaws contained higher levels of CD45+ leukocytes 1 and 3 d following i.d. CFA as compared with contralateral paws in all three conditions (WT, Ca_V_2.2^−/−^, and WT + i.d. ⍵-CgTx MVIIA; Fig. 2*f–k*). Overall CD45+ leukocyte infiltration associated with i.d. CFA therefore occurs independent of Ca_V_2.2 activity consistent with the importance of CD45+ leukocytes in edema (Ghasemlou et al., 2015), a process that is also Ca_V_2.2 channel independent (Fig. 2*a,b*).

**Figure 3.**
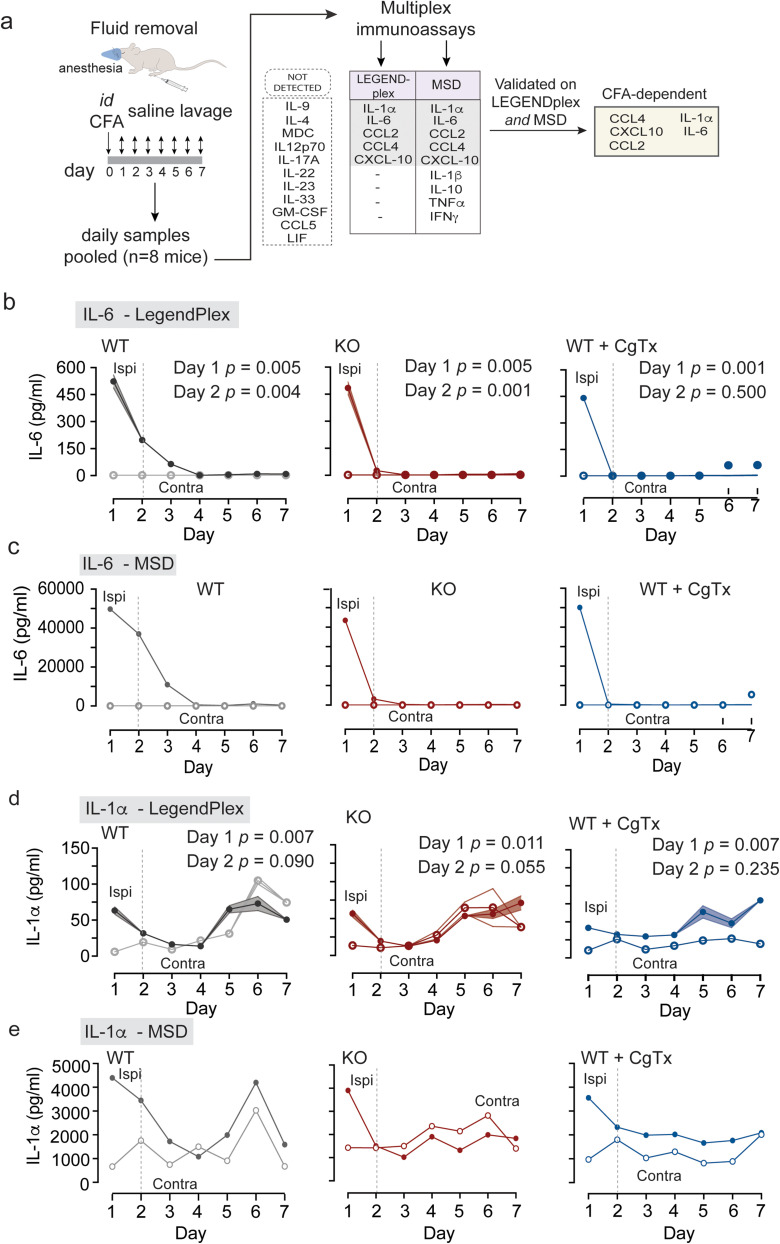
IL-6 levels in CFA-treated hindpaws are Ca_V_2.2 channel activity-dependent. IL-6 and IL-1ɑ cytokine levels measured daily for 1 week in contralateral and ipsilateral mouse hindpaw fluid using customized multiplex LEGENDplex and MSD immunoassays. Ipsilateral paw injected with 20 μl i.d. CFA in wild type (WT, gray), Ca_V_2.2^−/−^ (KO, red), and WT coinjected with 2 μM ⍵-CgTx MVIIA (WT + CgTx, blue). *N* = 8 mice for each condition. ***a***, The experimental protocol summarized: after the initial screen for 20 cytokines, 12 cytokines were analyzed in interstitial fluid collected daily from ipsilateral and contralateral hindpaws for three experimental conditions. Fluid from eight mice was pooled for each condition. Five cytokines were CFA-dependent and detected in both assays (IL-6, IL-1ɑ, CCL2, CCL4, and CXCL10; Extended Data Fig. 3-1). ***b***, ***d***, LEGENDplex analyses in technical triplicates of pooled samples from eight mice per condition. IL-6 (***b***) and IL-1ɑ (***d***) levels are elevated following CFA. IL-6 levels in ipsilateral hindpaws induced by CFA are Ca_V_2.2 channel dependent. ***b***, Values of IL-6 levels from ipsilateral hindpaw fluid are shown as mean ± SE. On Day 1: WT = 523 ± 36 pg/ml; Ca_V_2.2^−/−^ KO = 484 ± 34 pg/ml; WT + CgTx = 441 ± 16 pg/ml. On Day 2: WT = 197 ± 12 pg/ml; Ca_V_2.2^−/−^ KO = 25 ± 0.93 pg/ml; WT + CgTx = 1.2 ± 0.6 pg/ml. On Day 3: WT = 64 ± 15 pg/ml; Ca_V_2.2^−/−^ KO = 1.7 ± 0.1 pg/ml; WT + CgTx MVIIA = 1.17 ± 0.6 pg/ml. Analysis of variance using a repeated measures two-way ANOVA, followed by post hoc tests with Tukey multiple-comparisons correction (WT/KO/CgTx IL-6) | time interaction *p* < 0.0001. Day 2: *p*(WT ipsi | KO ipsi IL-6) = 0.0087; *p*(WT ipsi | WT CFA + CgTx MVIIA ipsi IL-6) = 0.0068. ***d***, Values of IL-1α levels from ipsilateral hindpaw fluid are shown as mean ± SE. On Day 1: WT = 63 ± 5.0 pg/ml; KO = 56 ± 4.8 pg/ml; WT + CgTx MVIIA = 42 ± 3.1 pg/ml. On Day 2: WT = 32 ± 1.4 pg/ml; KO = 19 ± 0.2 pg/ml; WT + CgTx MVIIA = 22 ± 11 pg/ml. On Day 3: WT = 16 ± 3.8 pg/ml; KO = 12 ± 0.3 pg/ml; WT + CgTx MVIIA = 30 ± 3.5 pg/ml. Analysis of variance using a repeated measures two-way ANOVA, followed by post hoc tests with Tukey multiple-comparisons correction (WT/KO/CgTx IL-1ɑ) | time interaction *p* = 0.0001. Day 2: *p*(WT ipsi | KO ipsi IL-1ɑ) = 0.0199; *p*(WT ipsi | WT CFA + CgTx MVIIA ipsi IL-1ɑ) = 0.6899. ***c***, ***e***, Electrochemiluminescence multiplex spot–based immunoassay (MSD custom R-PLEX, U-PLEX) validation of IL-6 (***c***) and IL-1ɑ (***e***) levels in the same samples used in ***b*** and ***d***. Mean values are from two technical replicates.

Further analysis of CD45+ leukocyte subtypes induced by i.d. CFA including monocytes, macrophages, Langerhans cells, and neutrophils (Fig. 2*f–k*; see also Materials and Methods) revealed increased neutrophils in paw punch biopsies of Ca_V_2.2^−/−^ KO and in WT mice coinjected ⍵-CgTx MVIIA as compared with WT mice (Fig. 2*d,g*) on Day 1 [*p*(WT CFA ipsi | KO CFA ipsi) = 0.00054; *p*(WT CFA ipsi | WT CFA + CgTx ipsi) = 0.0191; *p*(KO CFA ipsi | WT CFA + CgTx ipsi) = 0.7612; analysis of variance using a two-way ANOVA, followed by post-hoc tests with Tukey multiple-comparisons correction]. No other differences were observed based on the ANOVA (Extended Data Table 2-1). These data suggest that while not influencing CD45+ leukocyte infiltration overall, Ca_V_2.2 channel activity in the skin reduces neutrophil infiltration on Day 1 following i.d. CFA (Fig. 2*f–h*). Cytokines are released by both skin resident immune cells and infiltrating immune cells (Ellis and Bennett, 2013; Ghasemlou et al., 2015; Nguyen and Soulika, 2019). To further characterize changes in the inflammatory environment that are dependent on Ca_V_2.2 channel activity, we measured cytokine levels in hindpaws from pooled lavage fluid extracted daily (Fig. 3).

10.1523/ENEURO.0311-24.2024.f3-1 Figure 3-1 Cytokine levels in contralateral (control) and ipsilateral hind paws of mice measured daily for 1 week following 20 μL intradermal (*id*) injection of CFA in wildtype (WT, gray), CFA in Ca_V_2.2^-/-^ (KO, red), and CFA in WT co-injected with 2 μM ⍵-CgTx MVIIA (WT + CgTx, blue). N = 8 mice for each condition. Cytokine detection was performed using both LEGENDplex and MSD assays. **MSD SUPPLEMENT:** Electrochemiluminescence multiplex spot-based immunoassay (MSD R-Plex, U-plex) validation unveiled four additional cytokines: IL-1β, TNF-α, IFNγ, and IL-10 in paw lavage fluid. Samples were measured in technical replicates. In addition to the five cytokines IL-6, IL-1α**,** CCL2, CCL4, and CXCL10, the MSD platform detected IL-1β, TNF-α IFNγ, and IL-10 in paw lavage fluid. **LEGENDplex SUPPLEMENT**: Samples were measured in technical triplicates. Mean ± SE levels of CCL2 in ipsilateral paws on day 1 WT = 224 ± 17 pg/ml; KO = 258 ± 7.7 pg/ml; WT + CgTx MVIIA = 80 ± 4.6 pg/ml; day 2 WT = 92 ± 4.1 pg/ml; KO = 19 ± 0.9 pg/ml; WT + CgTx MVIIA = 17.5 ± 0.1 pg/ml; day 3: WT = 74 ± 14.2 pg/ml; KO = 56 ± 3.9 pg/ml; WT + CgTx MVIIA = 71.1 ± 5.1 pg/ml. p(WT/ KO/ CgTx MVIIA) | time interaction p < 0.0001. Analysis of variance of ipsilateral paw measured using two-way ANOVA with Tukey HSD correction for multiple comparisons and repeated measures. Mean ± SE levels of CCL4 in ipsilateral paws on day 1 WT = 29.4 ± 2.2 pg/ml; KO = 41.6 ± 3.5 pg/ml; WT + CgTx MVIIA = 16 ± 1.8 pg/ml; day 2 WT = 8.2 ± 0.4 pg/ml; KO = 8.7 ± 0.3 pg/ml; WT + CgTx MVIIA = 5.1 ± 0.5 pg/ml; day 3: WT = 7.0 ± 1.3 pg/ml; KO = 6.3 ± 0.3 pg/ml; WT + CgTx MVIIA = 11.6 ± 1.8 pg/ml. p(WT/ KO/ CgTx MVIIA) | time interaction p < 0.0001. Analysis of variance of ipsilateral paw measured using two-way ANOVA. **f.** Mean ± SE levels of CXCL10 in ipsilateral paws on day 1 WT = 182.3 ± 117.4 pg/ml; KO = 188.6 ± 9.3 pg/ml; WT + CgTx MVIIA = 273.1 ± 13.2 pg/ml; day 2 WT = 134.5 ± 63.5 pg/ml; KO = 77.7 ± 1.3 pg/ml; WT + CgTx MVIIA = 176.3 ± 20.9 pg/ml; day 3: WT = 305 ± 36.8 pg/ml; KO = 441.1 ± 10.6 pg/ml; WT + CgTx MVIIA = 611.4 ± 105 pg/ml. p(WT/ KO/ CgTx MVIIA) | time interaction p = 0.0005. Analysis of variance of ipsilateral paw measured using two-way ANOVA with Tukey HSD correction for multiple comparisons. Download Figure 3-1, TIF file.

10.1523/ENEURO.0311-24.2024.f3-2 Figure 3-2 Combined IL-6 and IL-1a data from the LEGENDplex and MSD immunoassays by normalizing cytokine levels to the concentration detected on Day 1 in the ipsilateral CFA injected paw. Download Figure 3-2, TIF file.

10.1523/ENEURO.0311-24.2024.t3-1 Table 3-1 Literature implicating cytokines in maladaptive prolonged inflammation. Download Table 3-1, DOC file.

10.1523/ENEURO.0311-24.2024.t3-2 Table 3-2 12-plex for LEGENDplex and MSD immunoassays. After initial pilot screens, two custom panels were developed to assess 12 cytokines across two platforms, 10 of the same cytokines were assessed on both platforms, and 2 were unique to each custom panel (bold lavender- LEGENDplex, bold blue- MSD). Download Table 3-2, DOC file.

### CFA-dependent increases in proinflammatory cytokines and levels are influenced by Ca_V_2.2 channel activity

Previous studies have shown that Ca_V_2.2 channel activity is necessary to trigger elevated levels of the cytokine IL-1α in the capsaicin model of fast, reversible neuroinflammation in the skin (Salib et al., 2024). Here, we initially screened 20 cytokines in fluid from ipsilateral and contralateral hindpaws over a 7 d period and showed 9 cytokines were elevated in hindpaws treated with i.d. CFA (Fig. 3*a*). We used this information to select 12 cytokines to generate a custom panel to analyze fluid extracted from ipsilateral and contralateral mouse hindpaws in the i.d. CFA model in WT, global Ca_V_2.2^−/−^ knock-out (KO), and WT coinjected with the highly specific Ca_V_2.2 blocker ⍵-CgTx MVIIA (Fig. 3; Extended Data Tables 3-1 and 3-2).

Daily fluid samples were analyzed by two independent immunoassay platforms: multiplex bead–based (LEGENDplex; Fig. 3*b,d*; Extended Data Fig. 3-1) and electrochemiluminescence spot–based (MSD R-PLEX, U-PLEX; Fig. 3*c,e*; Extended Data Fig. 3-1). Using LEGENDplex, we identified elevated levels of 5 of 12 cytokines on at least 1 of 7 d following i.d. CFA: IL-1α, IL-6, CXCL10, CCL2, and CCL4 (Fig. 3*a*; Extended Data Fig. 3-1). The MSD immunoassay showed elevated levels of the same five cytokines detected by the LEGENDplex, in addition to TNF-α, IL-1β, IFNγ, and IL-10. Validating our findings from the LEGENDplex immunoassay on the MSD immunoassay, we showed that the MSD immunoassay has greater sensitivity in paw fluid for a subset of cytokines when compared with LEGENDplex (Fig. 3 and Extended Data Fig. 3-1). The flow cytometry bead–based assay and the electrochemiluminescence-based assay differ in their methods of detection which likely accounts for their different sensitivities. TNF-α, IL-1β, IFNγ, and IL-10 are well-established cytokines involved in CFA-induced arthritic models of inflammation in synovial fluid in joints and serum. Levels of TNF-α, IL-1β, IFNγ, and IL-10 in mouse hindpaws are likely lower than typically reported where available tissue and volume are more abundant. The 7 d time course of cytokine levels reported here is the most comprehensive analysis of mouse hindpaw interstitial fluid to date. We focused our analysis on IL-1α, IL-6, CXCL10, CCL2, and CCL4 which were validated in both immunoassays. Of these cytokines, IL-6 levels were consistently longer in WT compared with both Ca_V_2.2^−/−^ KO and i.d. ω-CgTx MVIIA conditions (Fig. 3*b,c*).

We therefore focused on IL-6, which was elevated in response to i.d. CFA, dependent on Ca_V_2.2 channel activity, detected and validated in both immunoassay platforms, and for which in vivo validated neutralizing antibodies were available. IL-6 levels were elevated on Days 1–3 following i.d. CFA in WT hindpaws. By comparison, in Ca_V_2.2^−/−^ KO and i.d. ω-CgTx MVIIA WT mice IL-6 was transiently elevated on Day 1 and nearly undetectable on Days 2 and 3 [Day 2: *p*(WT ipsi | KO ipsi IL-6) = 0.0087; *p*(WT ipsi | WT CFA + CgTx MVIIA ipsi IL-6) = 0.0068 (WT| KO| CgTx) | time interaction *p* < 0.0001; Fig. 3*b,c*; analysis of variance using a repeated measures two-way ANOVA, followed by post hoc tests with Tukey multiple-comparisons correction]. These data show that IL-6 levels were elevated in hindpaw fluid following i.d. CFA in two independent immunoassay platforms, and its time course was dependent on peripheral Ca_V_2.2 channel activity.

### IL-6 and IL-1ɑ neutralizing antibodies reduced behavioral responses to i.d. CFA

**Figure 4.**
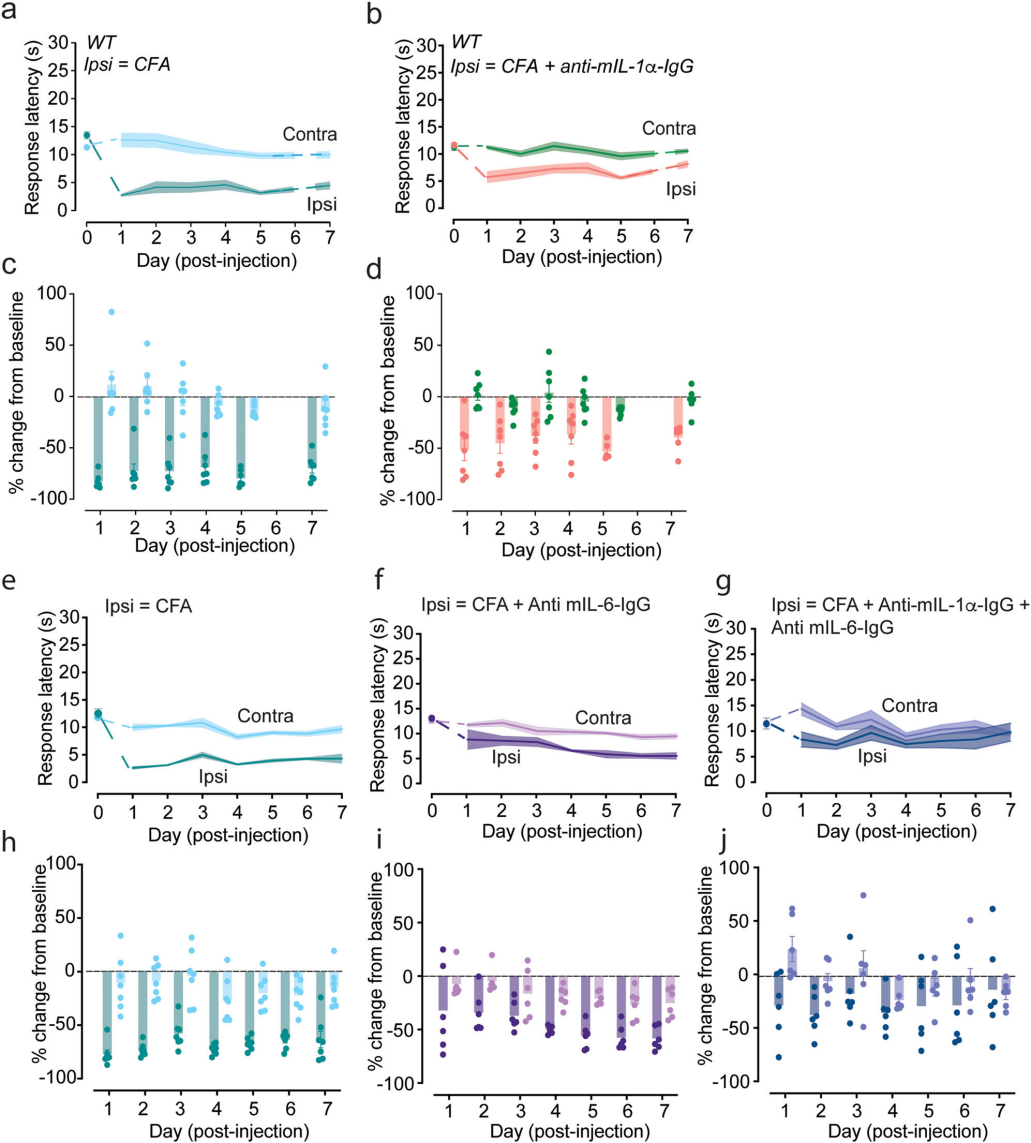
CFA-induced heat hypersensitivity was reduced in amplitude and time course by i.d. neutralizing antibodies to IL-6 and IL-1ɑ. ***a–j***, Withdrawal response latencies (s) to radiant heat in contralateral (contra) and ipsilateral (ipsi) paws shown as mean (lines) ± SE (shaded area; ***a***, ***b***, ***e–g***) and individual responses (solid circles) and mean values (shaded bars) as percent change from baseline (dotted line; ***c***, ***d***, ***h–j***). Measurements obtained immediately prior to (Day 0) and daily for 1 week after 20 μl i.d. CFA together with either saline (***a***, ***c***, ***e***, ***h***), 25 μg/ml anti-mIL-1α (***b***, ***d***), 100 μg/ml anti-mIL-6 (***f***, ***i***), or 25 μg/ml anti-mIL-1α + 100 μg/ml anti-mIL-6 (***g***, ***j***). ***a***, ***c***, Control CFA + saline, *n* = 7 (teal); (***b***, ***d***) CFA + 25 μg/ml anti-mIL-1α, *n* = 7 (salmon). Analysis of variance using a repeated measures two-way ANOVA, followed by post hoc tests with Tukey multiple-comparisons correction. Interaction between time | injection CFA, *p* = 0.0132; time | injection CFA + anti-mIL-1α, *p* = 0.2504. Day 3 *p*(WT CFA ipsi | contra) *p* < 0.0001, average percent change from baseline = −69.8%. Day 3 *p*(WT CFA + anti-mIL-1α ipsi | contra) *p* = 0.0036, average percent change from baseline = −40.9%. ***e***, ***h***, Control CFA + saline *n* = 7 (teal). ***f***, ***i***, CFA + 100 μg/ml anti-mIL-6, *n* = 6 (purple). ***g***, ***j***, CFA + 25 μg/ml anti-mIL-1α + 100 μg/ml anti-mIL-6, *n* = 6 (blue). Analysis of variance using a repeated measures two-way ANOVA, followed by post hoc tests with Tukey multiple-comparisons correction interaction between time | injection CFA, *p* = 0.0125; time | injection CFA + anti-mIL-6, *p* = 0.0427; and time | injection CFA + anti-mIL-1α + anti-mIL-6, *p* = 0.9181. Day 3 *p*(WT CFA ipsi | contra) *p* = 0.0008, average percent change from baseline = −58.33%. Day 3 *p*(WT CFA + anti-mIL-6 ipsi | contra) *p* = 0.0945, average percent change from baseline = −37.00%. Day 3 *p*(WT CFA + anti-mIL-1α + anti-mIL-6 ipsi | contra) *p* = 0.2613, average percent change from baseline = −13.80%.

To establish if there is a link between IL-6 and behavioral responses associated with i.d. CFA, we used anti-mIL-6-IgG (InvivoGen; anti-mIL-6-mIgG1e3 InvivoFit InvivoGen; catalog #mil6-mab15-1) coinjected with CFA (Fig. 4*f,i*) and compared the level of heat hypersensitivity to control animals (CFA alone, Fig. 4*e,h*). Anti-mIL-6-IgG reduced the development of heat hypersensitivity compared with control by 30–40% in the first 3 d following CFA (Fig. 4*f,i*). These data show that IL-6 is released in response to CFA and that it contributes directly to the development of heat hypersensitivity associated with prolonged neuroinflammation. Anti-mIL-6-IgG did not fully occlude CFA-induced heat hypersensitivity, consistent with the involvement of other cytokines in regulating neuronal responsiveness to sensory stimuli.

IL-1ɑ release occurs within 15 min of capsaicin-induced stimulation of heat-sensitive nociceptors (Salib et al., 2024), and rapid release in hindpaws was Ca_V_2.2 channel activity-dependent (Salib et al., 2024). Further, coinjection of mIL-1ɑ-IgG with capsaicin prevented the development of heat hypersensitivity (Salib et al., 2024). We therefore assessed the effect of anti-mIL-1ɑ-IgG (InvivoGen; anti-mIL-1α-mIgG1 InvivoFit; catalog #mil1a-mab9-1) in the CFA model of neuroinflammation and showed a reduction in the degree of heat hypersensitivity (Fig. 4*b,d*). Finally, we showed that the combination of both neutralizing monoclonal antibodies, anti-mIL-1ɑ-IgG and anti-mIL-6-IgG (Fig. 4*g*), was more effective at reducing the i.d. CFA-associated heat hypersensitivity compared with either anti-mIL-1ɑ-IgG (Fig. 4*b*) or anti-mIL-6-IgG alone (Fig. 4*f*); by Day 3, there was no significant difference in heat hypersensitivity in ipsilateral and contralateral paws (Days 3–7; injection | time interaction *p* = 0.5139; analysis of variance using a repeated measures two-way ANOVA, followed by post hoc tests with Tukey multiple-comparisons correction). These experiments show that both IL-1ɑ and IL-6 contribute to the induction and maintenance of CFA-induced heat hypersensitivity.

When combined with studies on IL-1ɑ which focus on the first 30 min of acute neuroinflammation following capsaicin exposure (Salib et al., 2024), we conclude that the release of IL-6 and IL-1ɑ is regulated by the activity of peripheral Ca_V_2.2 channels in acute transient and longer-lasting chronic forms of neuroinflammation in the skin.

## Discussion

Chronic neuroinflammation in the skin can follow peripheral nerve injury and prolonged exposure to damaging stimuli and is a precursor for a subset of neurodegenerative diseases (Ji et al., 2003; J. M. Zhang and An, 2007; Scholz and Woolf, 2007; Pinho-Ribeiro et al., 2017; B. C. Jiang et al., 2020). Hallmark behavioral responses of neuroinflammation in the skin include long-lasting hypersensitivity of sensory neurons to stimuli, lower response thresholds to noxious heat and mechanical stimuli, and perceiving previously innocuous sensory stimuli as painful (Costigan and Woolf, 2000; Scholz and Woolf, 2007; von Hehn et al., 2012; Ji et al., 2014). In addition, edema associated with increased leukocyte trafficking and extravasation into the injection site can develop (Schober, 2008) together with increased expression of signaling molecules that perpetuate inflammation triggered by chemokines including CCL2 and CXCL10 (Schober, 2008; Miller et al., 2009; Ghasemlou et al., 2015; Y. Chen et al., 2019; Prizant et al., 2021).

Many molecules including ion channels, neurotransmitters, and cytokines and their respective receptors are implicated in neuroinflammation that causes chronic pain (Extended Data Table 3-1; Costigan and Woolf, 2000; Scholz and Woolf, 2007; Basbaum et al., 2009; Pinho-Ribeiro et al., 2017; Trier et al., 2019; Jain et al., 2020; Tamari et al., 2021). In this study, we identify neuroinflammatory processes and molecules that depend on the activity of voltage-gated Ca_V_2.2 channels. Ca_V_2.2 channels are enriched in*Trpv1* nociceptors where they regulate synaptic transmission in the spinal cord (White and Cousins, 1998; Pitake et al., 2019) and in skin nerve endings (DuBreuil et al., 2021). Here, we provide direct evidence that peripheral voltage-gated Ca_V_2.2 channels and locally released cytokines, including IL-6, are key mediators of prolonged heat hypersensitivity that develops in response to neuroinflammation in the skin. Our experimental approach was designed to localize the site of action of cytokines and Ca_V_2.2 channels to the same hindpaw region where behavioral responses to radiant heat and mechanical stimulation were measured directly. We employed two independent multiplex cytokine assays to confirm the presence of cytokines, including IL-6, in hindpaw fluid during the first 3 d after CFA injection, when behavioral changes develop rapidly and are maximal.

### Peripheral Ca_V_2.2 channels—specific role in chronic heat hypersensitivity

Previous studies have shown that peripheral Ca_V_2.2 channels are critical for the development of heat hypersensitivity in the skin induced by intradermal capsaicin (DuBreuil et al., 2021); a model of rapid, adaptive increases in sensitivity to sensory stimuli in response to potentially damaging events that peak within 15 min and reverse within 30 min (DuBreuil et al., 2021). Here, we show that peripheral Ca_V_2.2 channels have a qualitatively similar role in CFA-induced heat hypersensitivity in the skin—which develops rapidly, but with a time course that lasts for days and involves ongoing release of several cytokines (Larson et al., 1986; Stein et al., 1988; Pitake et al., 2019). A single intradermal injection of ⍵-CgTx MVIIA, coincident with CFA injection, was highly effective at reducing the magnitude and shortening the time course of CFA-induced heat hypersensitivity (Fig. 1*e,f*; Extended Data Fig. 1-1). This suggests that early intervention can be highly effective at reducing the magnitude and time course of the neuroinflammatory response and that inhibition of peripheral Ca_V_2.2 channels is highly effective at curtailing heat hypersensitivity.

Mice that completely lack Ca_V_2.2 channels fail to develop heat hypersensitivity in response to i.d. CFA (KO; Fig. 1*b*), but these same mice develop mechanical hypersensitivity (Fig. 1*d*) and edema (Fig. 2*a,b*) at levels that were indistinguishable from WT. These data demonstrate that the neuroimmune signaling molecules that alter *Trpv1* nociceptor function diverge early in the inflammatory response from those that couple to mechanoreceptors. Our findings are consistent with several behavioral studies that point to the presence of distinct signaling pathways inducing heat and mechanical hypersensitivity (Fuchs et al., 2001; Sandkuhler, 2009; Y. Liu and Ma, 2011; Ebbinghaus et al., 2012; Usoskin et al., 2015; Ghitani et al., 2017), although they differ in this regard from White and Cousins (White and Cousins, 1998) who showed that daily injections of ⍵-CgTx MVIIA attenuated mechanical hypersensitivity in a peripheral nerve injury model of chronic pain (White and Cousins, 1998).

Mechanoreceptors express voltage-gated calcium channels that are distinct from heat-sensitive nociceptors (Francois et al., 2015; Cai et al., 2021; Hoppanova and Lacinova, 2022). In particular, Ca_V_3.2 channels (T-type currents) are expressed at high levels in a class of low-threshold mechanoreceptors (Walcher et al., 2018; Sharma et al., 2020; Jung et al., 2023) and have been implicated in CFA-induced mechanical hypersensitivity (Watanabe et al., 2015; Cai et al., 2021; Picard et al., 2023). Pharmacological and genetic manipulations of Ca_V_3.2 channels reduce CFA-induced mechanical hypersensitivity and reduce CFA-induced edema. Interestingly, levels of IL-6 were also reduced under conditions of reduced Ca_V_3.2 channel activity (Picard et al., 2023). This suggests that IL-6 signaling can be reduced by targeting Ca_V_3.2 and Ca_V_2.2 calcium ion channels and is consistent with studies showing that IL-6 has multiple peripheral cellular targets in the skin (Brenn et al., 2007; Summer et al., 2008; Pinho-Ribeiro et al., 2017).

The presence of CFA-induced edema in Ca_V_2.2^−/−^ KO mice that failed to develop heat hypersensitivity indicates that CFA is still triggering other neuroinflammatory signaling molecules responsible for edema and mechanical hypersensitivity. Interestingly, CFA-induced edema and heat hypersensitivity were also found to depend differentially on the gp130 protein, a signal transducer that complexes with IL-6Rs (Andratsch et al., 2009). Genetically silencing gp130 in small Na_V_1.8/*SNS*-expressing nociceptors (*SNS-gp130*^−/−^), a population of nociceptors that overlaps substantially with *Trpv1* nociceptors (Andratsch et al., 2009; Sharma et al., 2020; Jung et al., 2023), reduced CFA-induced heat hypersensitivity, while edema remained intact (Andratsch et al., 2009). This report parallels our findings implicating IL-6 signaling specifically in the development of heat hypersensitivity during CFA-induced neuroinflammation (Andratsch et al., 2009).

There is a difference in the time course of IL-6 levels compared with the development of heat hypersensitivity. Specifically, IL-6 levels fall to nondetectable levels by day 4 post-CFA injection whereas the behavioral response persists for at least 7 d. While we focus on IL-6 because its levels and time course depend on CFA and Ca_V_2.2 channel activity, we cannot exclude the possibility that other cytokines work in concert with IL-6 including CCL2, which has a similar timecourse, and could be contributing to signaling pathways responsible for prolonging heat hypersensitivity (Menetski et al., 2007; X. M. Wang et al., 2009; Van Steenwinckel et al., 2015; Dansereau et al., 2021). It is also highly likely that IL-6 acts on multiple downstream targets including immune cells which release other cytokines to prolong the inflammatory response (Murphy et al., 1999; Hurst et al., 2001; Andratsch et al., 2009; Hung et al., 2017; Huehnchen et al., 2020). Extended Data Figure 4 shows that levels of cytokines including CCL4, CXCL10, INFγ, and IL-10 are elevated in CFA-treated hindpaw, relative to the contralateral paw, through day 7. It is also possible that other signaling molecules not measured in our studies could contribute particularly to the later phase of the immune response to CFA. It will be interesting to establish whether the later phase of the inflammatory process is Ca_V_2.2 channel independent, reminiscent of the different phases of long-term synaptic plasticity which requires coincident calcium signals for initiation, but later signaling is independent of calcium (Ji et al., 2003; Lisman et al., 2012).

We also show that neutralizing IL-1ɑ and IL-6 together is more effective than either alone at preventing CFA-induced heat hypersensitivity implicating IL-1ɑ in the early inflammatory process triggered by CFA. This is also consistent with our previous studies which demonstrated elevated levels of IL-1ɑ in hindpaw fluid in response to capsaicin within 15 min, and the behavioral response is reduced by neutralizing IL-1α (Salib et al., 2024). Interestingly IL-1ɑ is known to stimulate the release of IL-6 suggesting that IL-1ɑ also contributes to the CFA inflammatory process (Orjalo et al., 2009). This is supported by the greater inhibitory effect on CFA-induced heat hypersensitivity of neutralizing IL-6 and IL-1ɑ simultaneously (Fig. 4*g*).

Elevated levels of IL-6 decline more rapidly in the absence of Ca_V_2.2 (KO) or following CgTx treatment, compared with WT hindpaw samples. But levels of IL-6 in hindpaw fluid on Day 1 were not very different across conditions. Fluid sample analysis started on Day 1 following CFA treatment, but it is likely that the peak IL-6 response occurred prior to day 1 when it is possible that IL-6 levels differ across conditions. We also know that IL-6 is released from multiple sources that are Ca_V_2.2-dependent and Ca_V_2.2-independent whereas our measurements are of bulk IL-6 levels in hindpaw. IL-1ɑ levels in mouse hindpaws following capsaicin treatment were also only partially inhibited by intradermal CgTx to block Ca_V_2.2 channels (Salib et al., 2024). It will be important to identify the target of IL-6 that underlies the heat hypersensitivity response of sensory nerve endings, to determine the source of IL-6, and whether its site of release is proximal to its functional target.

We also observed an increase of infiltrating leukocytes in CFA-injected paws, and this cellular response was independent of Ca_V_2.2 channel activity, consistent with intact edema. However, in hindpaws of global Ca_V_2.2^−/−^ KO mice and in WT hindpaws treated with ⍵-CgTx MVIIA, we measured a transient increase in neutrophil infiltration 1 d after i.d CFA (Fig. 2*d,g*). Additional experiments would be required to establish whether this change is functionally relevant, but a transient upregulation of neutrophils in an inflammatory response has been proposed to protect against the transition from acute to chronic pain in mice (Parisien et al., 2022). This is proposed to reduce the development of sensory neuron hypersensitivity, potentially through the secretion of endogenous opioids (Brack et al., 2004; Rittner et al., 2009; Diatchenko et al., 2022; Parisien et al., 2022).

### Cytokines dependent on Ca_V_2.2 channel activity

Sensory nociceptors are the target of a number of proinflammatory cytokines IL-1ɑ/IL-1β, IL-6, and TNF-α which act through their respective receptors IL-1R1, gp130, and TNFR1, respectively (Cunha et al., 2005; Ebbinghaus et al., 2012; D. Fang et al., 2015; Pinho-Ribeiro et al., 2017). We showed that IL-1ɑ modulates the excitability of TRPV1-expressing sensory neurons (Salib et al., 2024) adding to evidence that these cytokines and their receptors can mediate rapid changes in neuronal excitability associated with neuroinflammation including hypersensitivity to sensory stimuli (Brenn et al., 2007; Kanai et al., 2007; Barabas and Stucky, 2013; Malsch et al., 2014; A. D. Cook et al., 2018; Trier et al., 2019; Tamari et al., 2021). IL-1β (Safieh-Garabedian et al., 1995; Binshtok et al., 2008) and TNF-α (Tyagi et al., 2024) have been shown to act through effects on voltage-gated sodium and calcium channels (Binshtok et al., 2008; Picard et al., 2023; Tyagi et al., 2024). Here, we show that the inhibitory effects of simultaneously neutralizing IL-1ɑ and IL-6 cytokines in hindpaw on behavioral responses induced by CFA are similar in magnitude and time course to the intradermal block of Ca_V_2.2 channels by CgTx (compare Figs. 1*f*, 4*g*). But, to our knowledge, cytokines have not yet been shown to act directly on Ca_V_2.2 channels, and further studies are needed to establish the precise mechanism of action of IL-6 and other cytokines in altering sensory neuron excitability

 IL-6 has been shown by others to have an important role in the development of acute inflammatory pain (Murphy et al., 1999; Hurst et al., 2001; Summer et al., 2008; Andratsch et al., 2009; X. M. Wang et al., 2009; Wei et al., 2013; Malsch et al., 2014) and in modulating the excitability of sensory neuron excitability (X. M. Wang et al., 2009; Wei et al., 2013; D. Fang et al., 2015; Dansereau et al., 2021). We show that the CFA-induced time course for IL-6 depends on the activity of Ca_V_2.2 channels. Local levels of IL-6 in hindpaw fluid induced by CFA were greatly reduced on day 2 and eliminated on day 3, in hindpaws injected with ⍵-CgTx MVIIA and in Ca_V_2.2^−/−^ KO mice (Fig. 3*b,c*). Our discovery links Ca_V_2.2 channel activation with IL-6 signaling, adding important information about the key neuronal signals that initiate immune cell activation and perpetuate neuroinflammation.

To move beyond correlative observations and to establish a direct link between cytokine signaling and behavioral hypersensitivity, we used in vivo validated neutralizing antibodies to assess behavior affected by local IL-6 signaling. Compared to most studies, which delivered IL-6 neutralizing antibodies or IL-6 receptor antagonists via systemic or intrathecal routes, here we show that local hindpaw i.d. injection of anti-mIL-6-IgG is highly effective at reducing the degree of CFA-induced heat hypersensitivity. Our results highlight the importance of local cytokine signaling in the development and maintenance of ongoing neuroinflammation. This is consistent with our previous studies of capsaicin-induced heat and mechanical hypersensitivity which were inhibited by intradermal application of a neutralizing antibody to IL-1ɑ (Salib et al., 2024). These findings suggest that local ongoing inflammatory signaling in the skin can be interrupted in the periphery, without the need for central or systemic level intervention (White and Cousins, 1998; Lee et al., 2019; DuBreuil et al., 2021; Salib et al., 2024).

**Figure 5.**
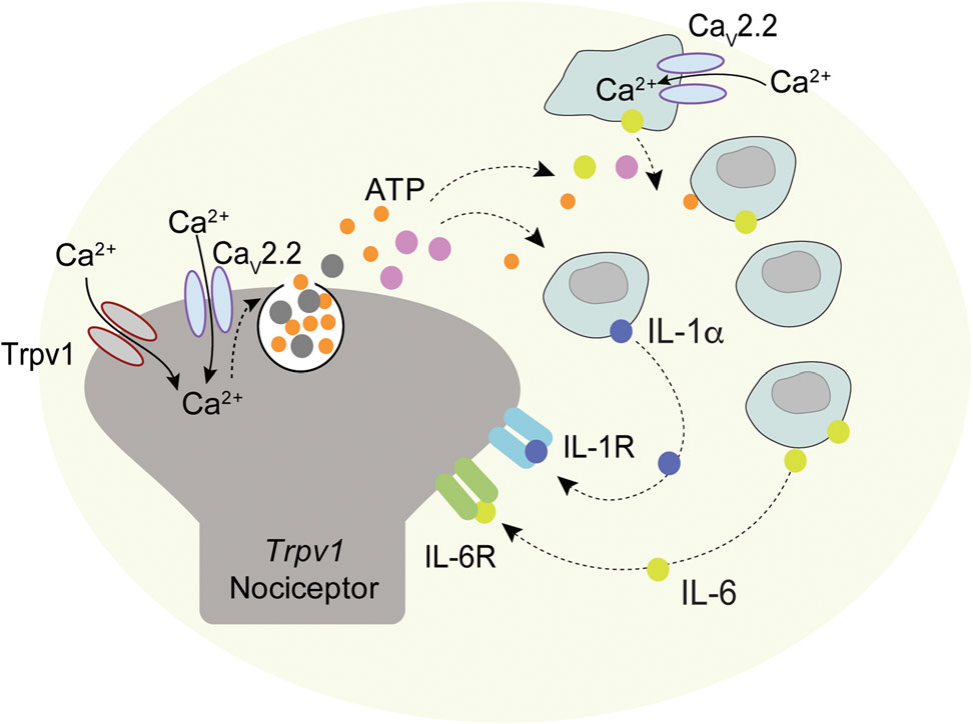
The model focused on the role of IL-6, IL-1ɑ, and Ca_V_2.2 channels in CFA-induced neuroinflammatory signaling. Ca_V_2.2 channels are enriched in *Trpv1* nociceptors including in nerve endings in the skin (DuBreuil et al., 2021). *Trpv1* nociceptors depolarize in response to inflammatory agents including CFA. Depolarization results in Ca_V_2.2 channel activation, triggering the release of proinflammatory neuropeptides and ATP, and these transmitters can signal to resident and infiltrating immune cells to release cytokines including IL-6 and IL-1ɑ. Others have shown immune cells can express voltage-gated ion channels (Feske et al., 2015; Picard et al., 2023) including Ca_V_2.2 (Saegusa et al., 2001; Huntula et al., 2019), and ATP can be released by immune cells in response to tissue injury or insults and can undergo autocrine purinergic signaling (Junger, 2011; Eltzschig et al., 2012). Increased levels of IL-6 and IL-1ɑ in hindpaws act via IL-6R and IL-1R expressed in *Trpv1* nociceptors (Andratsch et al., 2009; Schaible, 2014; D. Fang et al., 2015; Martin et al., 2021). IL-6 and IL-1ɑ activation of *Trpv1* nociceptors is linked to hypersensitivity to heat associated with both acute and long-lasting neuroinflammation models (Brenn et al., 2007; Andratsch et al., 2009; Wei et al., 2013; Malsch et al., 2014; D. Fang et al., 2015; Huehnchen et al., 2020; Jeevakumar et al., 2020). Reducing or eliminating Ca_V_2.2 channel activity (Fig. 1) or neutralizing the actions of IL-6 and or IL-1ɑ locally inhibits the magnitude and time course of CFA-induced heat hypersensitivity (Fig. 4).

Multiple cytokines contribute to ongoing neuroinflammation, but we also show that the combination of neutralizing IL-6 and IL-1ɑ resulted in attenuation of CFA-induced heat hypersensitivity by day 3 (Fig. 4*g*). While it is very likely that many other cytokines can play a role in CFA-induced neuroinflammation, we find that neutralizing only two of these cytokines is sufficient to significantly shorten the time course of heat hypersensitivity. Cell surface–bound IL-1α acts as an upstream regulator of IL-6 secretion, and depletion of IL-1α using a neutralizing antibody reduces the DNA binding activity of NF-kB which stimulates downstream IL-6 transcription (Orjalo et al., 2009). IL-1α is released within the first 15 min of neuroinflammation induced by intradermal capsaicin (Salib et al., 2024), and it is likely that IL-1α is a key upstream regulator of IL-6 release (Fig. 5). While the focus of this report is primarily on the role of Ca_V_2.2 in sensory neurons, others have reported the presence of voltage-gated ion channels in nonneuronal cells including T-cells and macrophages (Feske et al., 2015; Picard et al., 2023) as well as microglia (Saegusa et al., 2001; Huntula et al., 2019). It is possible that resident and infiltrating immune cells express voltage-gated ion channels that could also be targeted by a local block of Ca_V_2.2 channels (Fig. 5).

Our data presented here add new evidence that links the activation of peripheral voltage-gated Ca_V_2.2 channels to local cytokine signaling involved in the development and maintenance of heat hypersensitivity associated with prolonged neuroinflammatory pain. Chronic forms of neuroinflammation involve many signaling molecules that can mediate bidirectional communication between sensory neurons and immune cells. Here, we focus on the importance of peripheral Ca_V_2.2 channels localized to the site of inflammation and their critical role in the induction and maintenance of heat hypersensitivity that lasts for days. We also identify IL-6 as one of the several cytokines communicating between sensory neurons and immune cells to propagate inflammatory signaling underlying prolonged heat hypersensitivity. Importantly, we show that local inhibition of Ca_V_2.2 channels or neutralizing the actions of IL-1ɑ and IL-6, at the peripheral origin of neuroinflammation, are all highly effective at reducing the amplitude and time course of heat hypersensitivity in the skin. It is possible that other models of chronic inflammation including peripheral nerve injury and postoperative pain might recruit inflammatory mechanisms like those reported here. The importance of peripheral Ca_V_2.2 channels in the capsaicin model of rapidly developing and transient heat hypersensitivity and in CFA-induced week-long heat hypersensitivity raises the likelihood that these channels are important in other forms of chronic inflammation resulting from peripheral nerve injury. Future studies could test this hypothesis to assess the potential therapeutic benefits of blocking peripheral Ca_V_2.2 channels at sites of nerve damage.

## Synthesis

Reviewing Editor: Karl Herrup, University of Pittsburgh School of Medicine Decisions are customarily a result of the Reviewing Editor and the peer reviewers coming together and discussing their recommendations until a consensus is reached. When revisions are invited, a fact-based synthesis statement explaining their decision and outlining what is needed to prepare a revision will be listed below. The following reviewer(s) agreed to reveal their identity: Lulu Jiang.

Synthesis: eN-NWR-0311-24

Peripheral CaV2.2 channels in skin regulate prolonged heat hypersensitivity during neuroinflammation

The authors employ two approaches to investigate the role of CaV2.2 channels in mediating the effects of the Complete Freund's Adjuvant (CFA) model of chronic neuroinflammation. Their approach is both genetic (CaV2.2 mutant mice) and pharmacological (CaV2.2 blocker ⍵-CgTx MVIIA), and the consistency of the findings across experimental models provides strong validity to the model used. The results provide data supporting a model where CaV2.2 activity regulates skin-localized leukocyte profiles and cytokine levels following CFA insult. Their study reveals that CaV2.2 channel activity is associated with IL-6 release in the skin and plays a crucial role in inducing and maintaining heat hypersensitivity during chronic neuroinflammation. Thus, this study adds to our understanding by identifying CaV2.2, IL-6, and IL-1α as critical players of chronic neuroinflammation and heat hypersensitivity in a CFA model.

The findings are potentially important for the field, but several flaws detract significantly from the overall impact of the work.

The statistics need significant revisions to be acceptable. For example, the authors report differences between WT and CaV2.2-/- KO mice in baseline measures, but no statistical analyses or figures are presented. Without these the authors should rephrase the text to clarify that this difference is just a qualitative observation. In Figure 2 the statistical methods used are not specified (e.g., two-way ANOVA). Throughout, the authors should clarify if they ran a repeated measures ANOVA where it was appropriate. For clarity, p-values should be shown in panels Fig 2F-K. Similarly, in Figure 3 the statistical analyses used panels 3B-E must be specified and presented. As it is an important point of the paper, it is recommended that Figures 3A and 3B be combined for direct statistical comparison between WT and CFA with treatment. Similarly, Fig 4C and 4D should be combined for improved clarity.

Reorganization of the figures would clarify the authors' points. For example, comparisons between WT and KO could be shown in the same panel, with different cell types in separate panels. Figure 2F could display monocyte comparisons with two factors: control vs. lpsi and WT vs. KO vs. WT+CgTx. Figure 3 could also be re-organized so that WT and KO are in the same panel. In Figure 1, adding bar graphs (along with individual data points) to the % change data may depict the means better.

In Figure 2K, the number of macrophages, Langerhans cells, and neutrophils appear to be lower than the levels in 2I and 2J. Were there any statistical differences between groups in these measures? If so, might this explain the complete loss of heat hypersensitivity observed on day 3 in the CgTx group (Fig. 1f)? Some discussion to this effect may be appropriate.

In Figure 3, the elevated levels of cytokines in the WT group is eliminated by Day 4, yet the heat hypersensitivity is maintained up to Day 7. How might authors explain this? In the KO and CgTx groups, blocking CaV2.2 activity appears to expedite this elimination process by one or two days. However, the levels are similar on Day 1 across three groups. This temporal pattern may be worth discussing as it raises the question of whether CaV2.2 involvement in the development of heat hypersensitivity is delayed relative to the initial insult. Does this indicate that disengagement of CaV2.2 at the time of insult renders these channels insensitive to ongoing inflammation, thereby terminating the neuroimmune feedback cycle?

Some discussion is warranted concerning which intracellular signaling cascades lead to cytokine modulation of neuronal excitability. Is there any evidence that they may feed back into CaV2.2 modulation to continue the cycle?

Other minor corrections would further improve the submission.

On line 301 C57BL/6 mice are described as outbred, but they are inbred.

The authors wait until line 86 of the Introduction to state the main aim of the study. It might be more effective if it were unveiled earlier in the text. One possible alterntative: the sentence in line 86 could be better placed after line 79, followed by the introduction of the model used.

In the Introduction, the authors should clarify the order of events in the inflammatory process. On line 49, the authors seem to suggest that the rise in neuronal calcium initiates the neuroinflammatory cycle. The authors may want to point out other targets of the noxious stimuli such as the dendritic cells and other immune cells which may initiate or simultaneously commence the inflammatory process.

On line 84, authors may want to refer to CFA as a heat killed mycobacterium containing adjuvant, rather than the mycobacterium itself.

Please clarify why, in Figures 1C and D, the day 4 and 5 data are missing for Hargreaves test?

Figure 2B depicts data for three groups on the same plot. However, they appear collapsed because the measures are very similar across the groups. The authors might consider adding proper legends or adding separate figures for each treatment group or some other means of clarifying the presentation

In Figure 2 the authors claim differences between control and treatment groups but figures representing these direct comparisons are missing. It might also be worth considering the inclusion of a CD45+ pan-leukocytes data, alongside the leukocyte subtypes data (Figure 2F-K). Day 1 versus day 3 data group differences appear to exist in the change in levels between the two days and would benefit from deeper analysis as it could provide important insight into the temporal dynamics of the leukocyte profile.

Please state whether the order of behavioral testing counterbalanced between ipsi and contralateral side?

How many trials were conducted for baseline measure of Von Frey test? Was it three trials as it was for the Hargreaves test?

The authors might wish to discuss as future directions the relevance of the current study to a different model of chronic inflammation such as peripheral nerve injury.

The summary schematic is an asset to the paper. Reflecting on its implications, there is evidence, albeit sparse, that Cav2.2 in non-excitable cells like the microglia is functional in a pathophysiological state (Saegusa &Tanabe, 2014, Huntula...Tanabe el al., 2019). Authors might discuss an alternative pathway by which CaV2.2 may affect leukocyte and cytokine profile. Authors may consider including this information in their schematic.
