# Supplementary figures and images for: Peripheral CaV2.2 Channels in the Skin Regulate Prolonged Heat Hypersensitivity during Neuroinflammation
Source: eNeuro. 2024 Nov 19;11(11):ENEURO.0311-24.2024. doi: 10.1523/ENEURO.0311-24.2024 (PMC11599794; doi:10.1523/ENEURO.0311-24.2024)

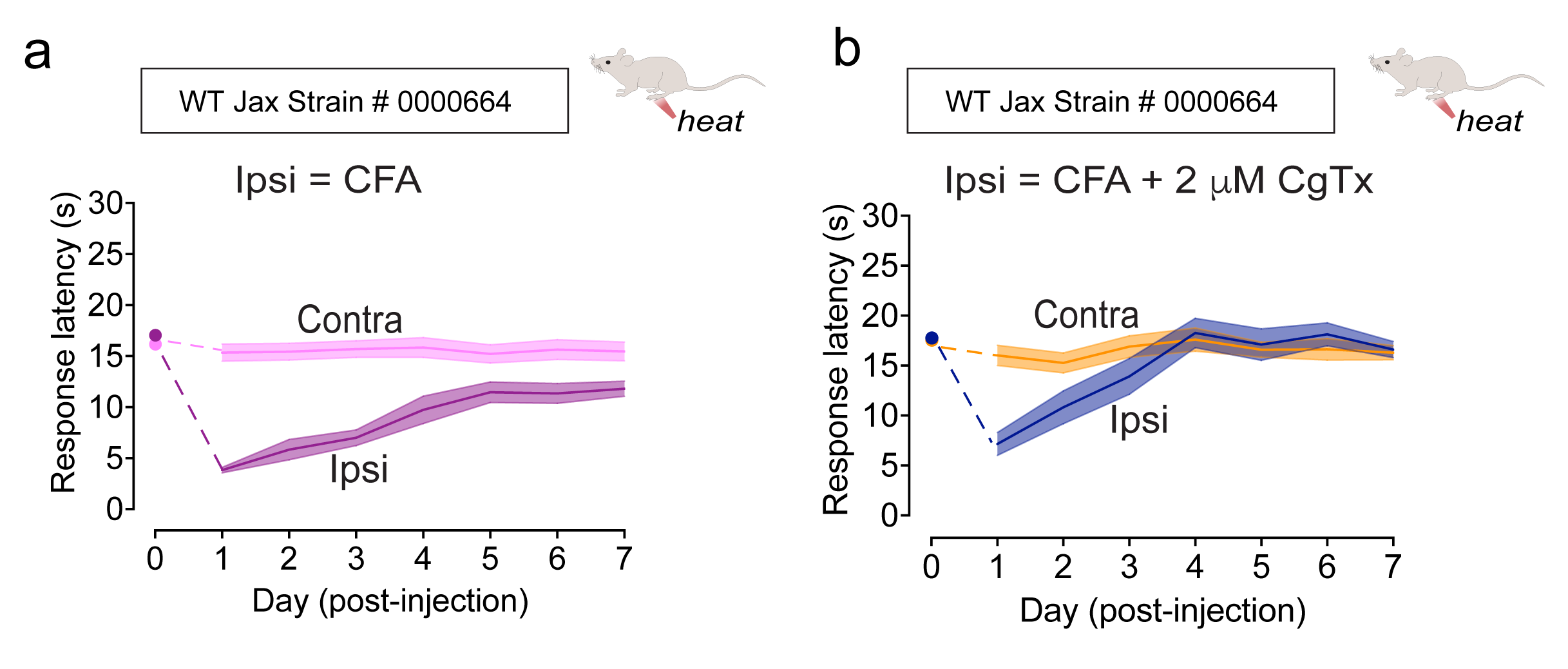

Supplement: Figure 1-1 — Validation of CFA-induced heat hypersensitivity inhibited by pharmacological block of peripheral CaV2.2 channels in an inbred independent mouse strain. 16–20-week-old C57BL/6J mice were ordered from Jackson Laboratories (Strain # 0000664) and were acclimated at least one week prior to behavioral testing. Under blinded conditions, mice were injected with CFA alone (n = 13), or CFA + 2 μM ⍵-CgTx MVIIA (n = 13) as described previously (see in methods and figure 2. Solid line represents the mean response latency, the shaded area represents standard error. Download Figure 1-1, TIF file. [file eneuro-11-ENEURO.0311-24.2024-s002.tif]

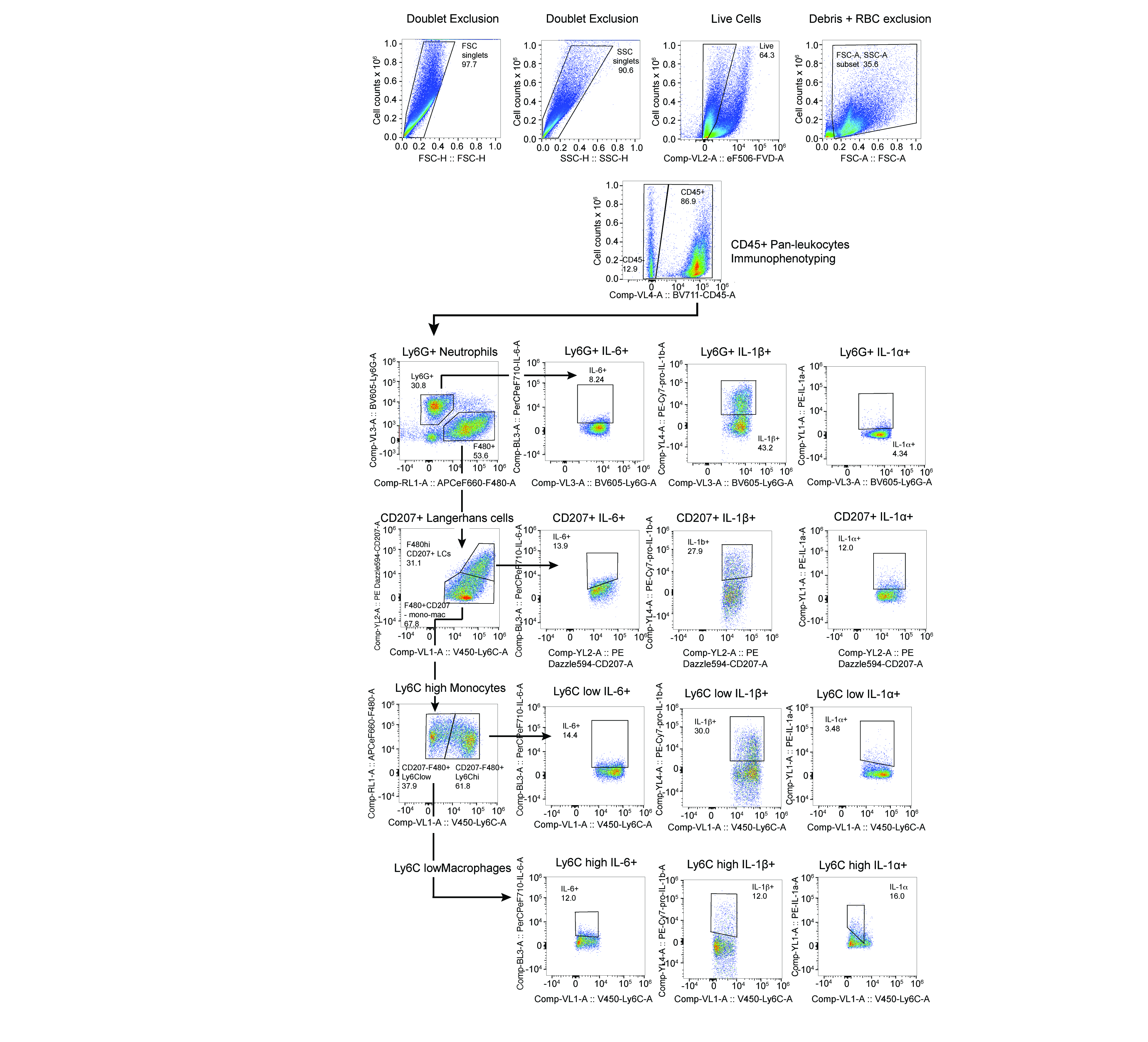

Supplement: Figure 2-1 — Gating strategy for hind paw deep punch biopsy immunophenotyping flow cytometry analyses (shown in Fig. 2) Analyses were performed using FlowJo Software v10.9.0. Housekeeping exclusion of cell doublets, dead cells, debris, and red blood cells. Cells were assigned an identity based on expression of specific cell surface markers using antibodies with distinct fluorophores. The cell surface marker CD45 was used to identify pan-leukocytes, that were further characterized into individual cell populations based on the expression of additional cell surface markers: Neutrophils express Ly6G, Langerhans cells express F4/80 and CD207, Monocytes express F4/80 and high expression of Ly6C, and Macrophages express F4/80 and low expression of Ly6C. For each leukocyte population, we analyzed intracellular cytokine levels of IL-6, IL-1β, and IL-1α using intracellular cytokine antibodies (See Table 1 and Methods). Download Figure 2-1, TIF file. [file eneuro-11-ENEURO.0311-24.2024-s003.tif]

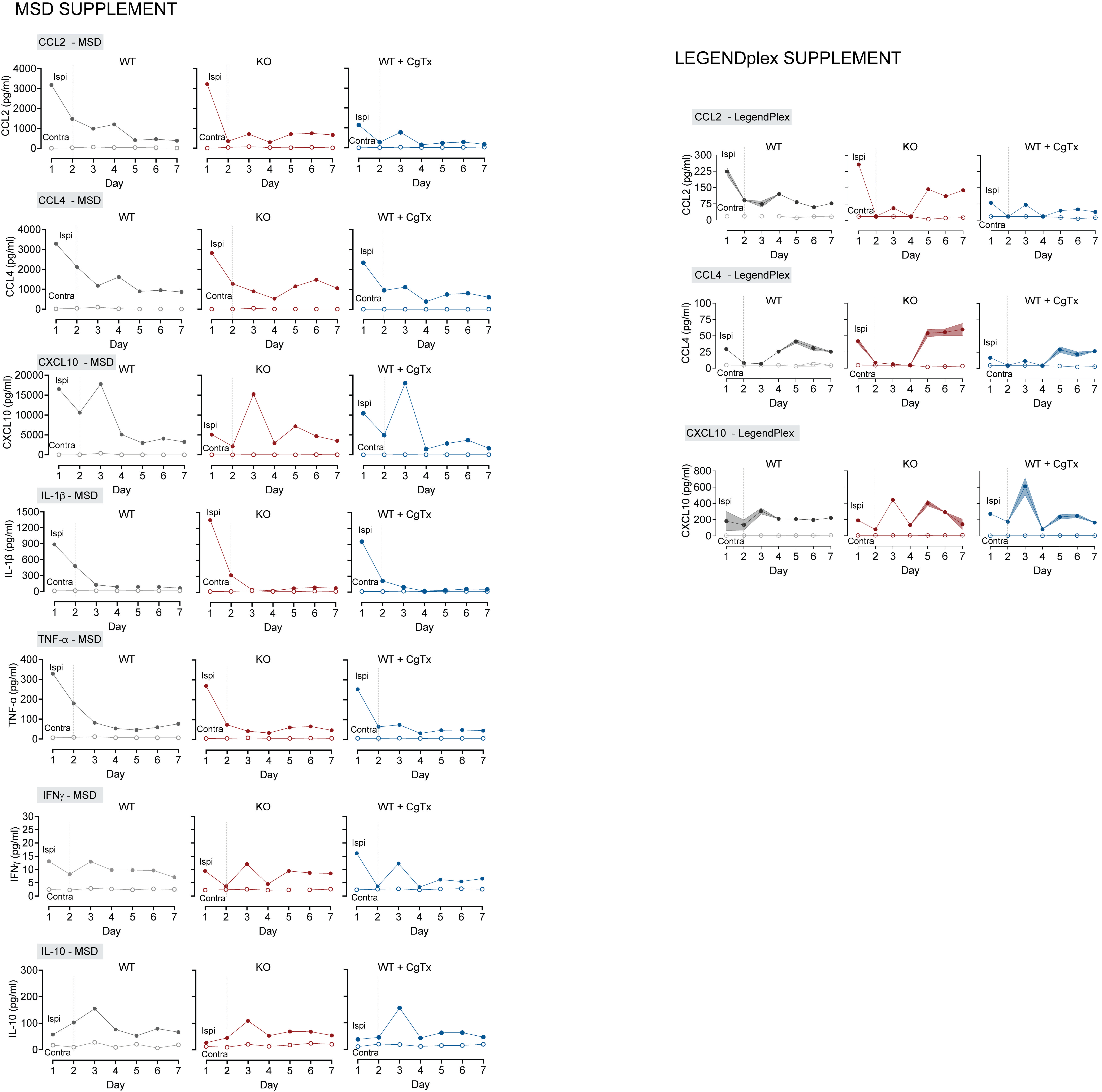

Supplement: Figure 3-1 — Cytokine levels in contralateral (control) and ipsilateral hind paws of mice measured daily for 1 week following 20 μL intradermal (id) injection of CFA in wildtype (WT, gray), CFA in CaV2.2-/- (KO, red), and CFA in WT co-injected with 2 μM ⍵-CgTx MVIIA (WT + CgTx, blue). N = 8 mice for each condition. Cytokine detection was performed using both LEGENDplex and MSD assays. MSD SUPPLEMENT: Electrochemiluminescence multiplex spot-based immunoassay (MSD R-Plex, U-plex) validation unveiled four additional cytokines: IL-1β, TNF-α, IFNγ, and IL-10 in paw lavage fluid. Samples were measured in technical replicates. In addition to the five cytokines IL-6, IL-1α, CCL2, CCL4, and CXCL10, the MSD platform detected IL-1β, TNF-α IFNγ, and IL-10 in paw lavage fluid. LEGENDplex SUPPLEMENT: Samples were measured in technical triplicates. Mean ± SE levels of CCL2 in ipsilateral paws on day 1 WT = 224 ± 17 pg/ml; KO = 258 ± 7.7 pg/ml; WT + CgTx MVIIA = 80 ± 4.6 pg/ml; day 2 WT = 92 ± 4.1 pg/ml; KO = 19 ± 0.9 pg/ml; WT + CgTx MVIIA = 17.5 ± 0.1 pg/ml; day 3: WT = 74 ± 14.2 pg/ml; KO = 56 ± 3.9 pg/ml; WT + CgTx MVIIA = 71.1 ± 5.1 pg/ml. p(WT/ KO/ CgTx MVIIA) | time interaction p < 0.0001. Analysis of variance of ipsilateral paw measured using two-way ANOVA with Tukey HSD correction for multiple comparisons and repeated measures. Mean ± SE levels of CCL4 in ipsilateral paws on day 1 WT = 29.4 ± 2.2 pg/ml; KO = 41.6 ± 3.5 pg/ml; WT + CgTx MVIIA = 16 ± 1.8 pg/ml; day 2 WT = 8.2 ± 0.4 pg/ml; KO = 8.7 ± 0.3 pg/ml; WT + CgTx MVIIA = 5.1 ± 0.5 pg/ml; day 3: WT = 7.0 ± 1.3 pg/ml; KO = 6.3 ± 0.3 pg/ml; WT + CgTx MVIIA = 11.6 ± 1.8 pg/ml. p(WT/ KO/ CgTx MVIIA) | time interaction p < 0.0001. Analysis of variance of ipsilateral paw measured using two-way ANOVA. f. Mean ± SE levels of CXCL10 in ipsilateral paws on day 1 WT = 182.3 ± 117.4 pg/ml; KO = 188.6 ± 9.3 pg/ml; WT + CgTx MVIIA = 273.1 ± 13.2 pg/ml; day 2 WT = 134.5 ± 63.5 pg/ml; KO = 77.7 ± 1.3 pg/ml; WT + CgTx MVIIA = 176.3 ± 20.9 pg/ml; da [file eneuro-11-ENEURO.0311-24.2024-s004.tif]

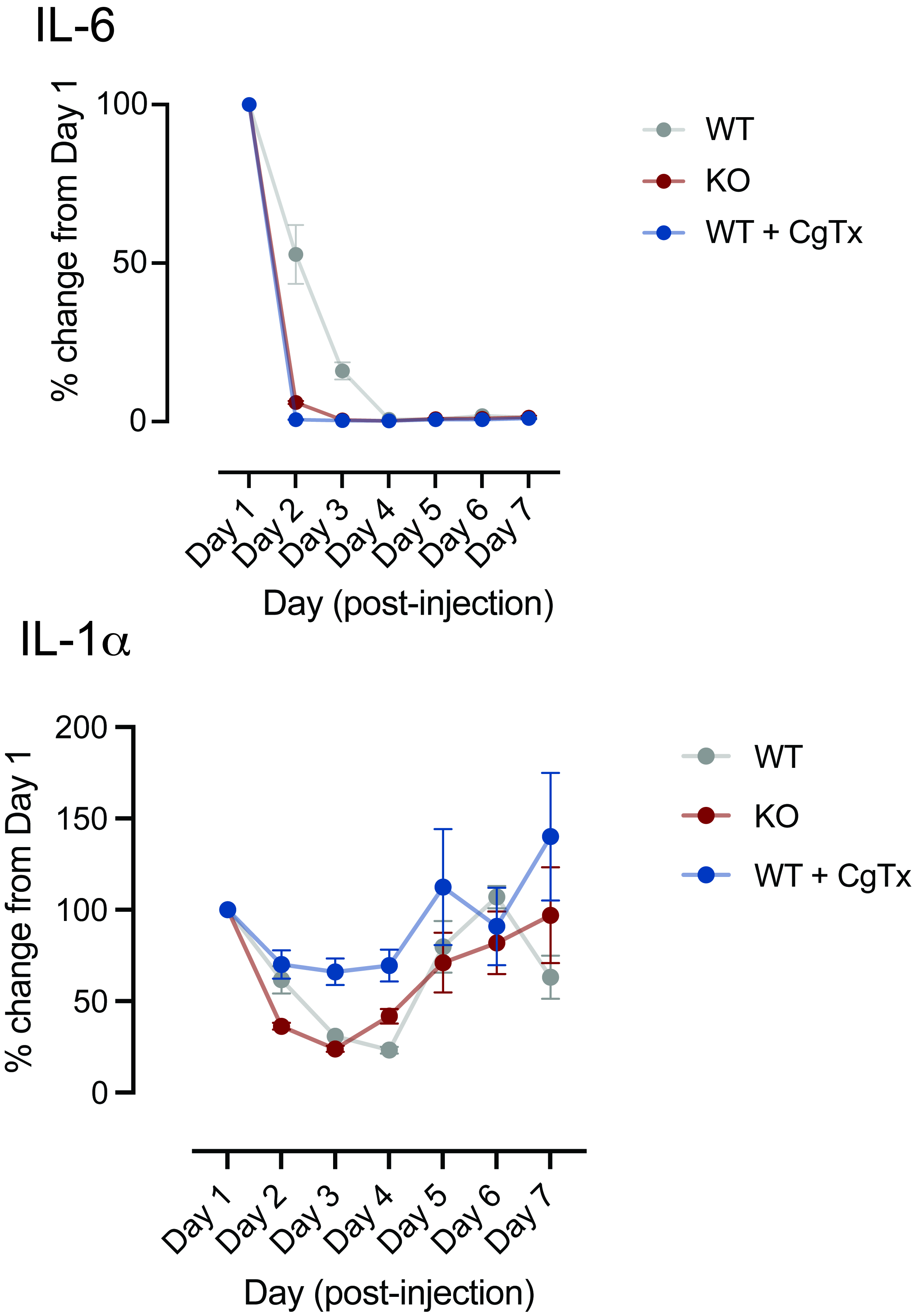

Supplement: Figure 3-2 — Combined IL-6 and IL-1a data from the LEGENDplex and MSD immunoassays by normalizing cytokine levels to the concentration detected on Day 1 in the ipsilateral CFA injected paw. Download Figure 3-2, TIF file. [file eneuro-11-ENEURO.0311-24.2024-s005.tif]
