# Supplementary material for: Peripheral CaV2.2 Channels in the Skin Regulate Prolonged Heat Hypersensitivity during Neuroinflammation
Source: eNeuro. 2024 Nov 19;11(11):ENEURO.0311-24.2024. doi: 10.1523/ENEURO.0311-24.2024 (PMC11599794; doi:10.1523/ENEURO.0311-24.2024)
Supplement: Table 2-1 — Statistical analysis of variance using 2-way ANOVA with Tukey HSD correction for multiple comparisons across conditions, cell types, and days, with adjusted p-values. Download Table 2-1, DOC file. [file eneuro-11-ENEURO.0311-24.2024-s006.doc]

| **Day 1** |  | **Day 3** | |
| --- | --- | --- | --- |
|  |  |  |  |
| **Monocytes** | **P values** | **Monocytes** | **P values** |
| WT vs KO | 0.6003 | WT vs KO | 0.9691 |
| WT vs WT + CgTx | 0.7763 | WT vs WT + CgTx | 0.9825 |
| KO vs WT + CgTx | 0.9331 | KO vs WT + CgTx | 0.9120 |
|  |  |  |  |
| **Langerhan Cells** | **P values** | **Langerhan Cells** | **P values** |
| WT vs KO | 0.9370 | WT vs KO | 0.9447 |
| WT vs WT + CgTx | 0.9357 | WT vs WT + CgTx | 0.5174 |
| KO vs WT + CgTx | 0.7727 | KO vs WT + CgTx | 0.3628 |
|  |  |  |  |
| **Macrophages** | **P values** | **Macrophages** | **P values** |
| WT vs KO | 0.9870 | WT vs KO | 0.7194 |
| WT vs WT + CgTx | 0.9941 | WT vs WT + CgTx | 0.9619 |
| KO vs WT + CgTx | 0.9980 | KO vs WT + CgTx | 0.5619 |
|  |  |  |  |
| **Neutrophils** | **P values** | **Neutrophils** | **P values** |
| WT vs KO | 0.0054 | WT vs KO | 0.9080 |
| WT vs WT + CgTx | 0.0191 | WT vs WT + CgTx | 0.3256 |
| KO vs WT + CgTx | 0.7612 | KO vs WT + CgTx | 0.1803 |

**Extended Data Table 2-1:** Statistical analysis of variance using 2-way ANOVA with Tukey HSD correction for multiple comparisons across conditions, cell types, and days, with adjusted p-values.
