# Supplementary material for: Peripheral CaV2.2 Channels in the Skin Regulate Prolonged Heat Hypersensitivity during Neuroinflammation
Source: eNeuro. 2024 Nov 19;11(11):ENEURO.0311-24.2024. doi: 10.1523/ENEURO.0311-24.2024 (PMC11599794; doi:10.1523/ENEURO.0311-24.2024)
Supplement: Table 3-1 — Literature implicating cytokines in maladaptive prolonged inflammation. Download Table 3-1, DOC file. [file eneuro-11-ENEURO.0311-24.2024-s007.doc]

| **Cytokine** | **References** |
| --- | --- |
| IL-1α | ;;;; |
| IL-1β | ;;;; |
| IL-4 | ;;; |
| IL-6 | ;; |
| IL-10 | ;; |
| TNF-α | ;; |
| LIF | ;; |
| CXCL10 | ;; |
| CCL2 | ;; |
| CCL4 | ;, |
| IFNγ | ;;; |
| MDC | ; |
| IL-33 | ; |

**Extended Data Table 3-1:** Literature implicating cytokines in maladaptive prolonged inflammation.
