# Supplementary material for: Peripheral CaV2.2 Channels in the Skin Regulate Prolonged Heat Hypersensitivity during Neuroinflammation
Source: eNeuro. 2024 Nov 19;11(11):ENEURO.0311-24.2024. doi: 10.1523/ENEURO.0311-24.2024 (PMC11599794; doi:10.1523/ENEURO.0311-24.2024)
Supplement: Table 3-2 — 12-plex for LEGENDplex and MSD immunoassays. After initial pilot screens, two custom panels were developed to assess 12 cytokines across two platforms, 10 of the same cytokines were assessed on both platforms, and 2 were unique to each custom panel (bold lavender- LEGENDplex, bold blue- MSD). Download Table 3-2, DOC file. [file eneuro-11-ENEURO.0311-24.2024-s008.doc]

| **LEGENDplex** | **MSD** |
| --- | --- |
| IL-1β | IL-1β |
| IFNγ | IFNγ |
| TNF-α | TNF-α |
| CCL4 | CCL4 |
| CCL2 | CCL2 |
| CXCL10 | CXCL10 |
| IL-6 | IL-6 |
| IL-1α | IL-1α |
| IL-10 | IL-10 |
| IL-23 | IL-23 |
| **LIF** | **MDC** |
| **IL-4** | **IL-33** |

**Extended Data Table 3-2:** 12-plex for LEGENDplex and MSD immunoassays. After initial pilot screens, two custom panels were developed to assess 12 cytokines across two platforms, 10 of the same cytokines were assessed on both platforms, and 2 were unique to each custom panel (bold lavender- LEGENDplex, bold blue- MSD).
